# Alternative splicing of the Snap23 microexon is regulated by MBNL, QKI, and RBFOX2 in a tissue-specific manner and is altered in striated muscle diseases

**DOI:** 10.1080/15476286.2025.2491160

**Published:** 2025-04-10

**Authors:** Gabrielle M. Gentile, R. Eric Blue, Grant A. Goda, Bryan B. Guzman, Rachel A. Szymanski, Eunice Y. Lee, Nichlas M. Engels, Emma R. Hinkle, Hannah J. Wiedner, Aubriana N. Bishop, Jonathan T. Harrison, Hua Zhang, Xander H.T. Wehrens, Daniel Dominguez, Jimena Giudice

**Affiliations:** a Department of Cell Biology and Physiology, The University of North Carolina at Chapel Hill, Chapel Hill, NC, USA; b Curriculum in Genetics and Molecular Biology, The University of North Carolina at Chapel Hill, Chapel Hill, NC, USA; c Department of Chemistry, The University of North Carolina at Chapel Hill, Chapel Hill, NC, USA; d Department of Pharmacology, The University of North Carolina at Chapel Hill, Chapel Hill, NC, USA; e Cardiovascular Research Institute, Baylor College of Medicine, Houston, TX, USA; f Lineberger Comprehensive Cancer Center, The University of North Carolina at Chapel Hill, Chapel Hill, NC, USA; g RNA Discovery Center, The University of North Carolina at Chapel Hill, Chapel Hill, NC, USA; h McAllister Heart Institute, The University of North Carolina at Chapel Hill, Chapel Hill, NC, USA

**Keywords:** Microexon, alternative splicing, striated muscle, Snap23, RNA-binding proteins

## Abstract

The reprogramming of alternative splicing networks during development is a hallmark of tissue maturation and identity. Alternative splicing of microexons (small, genomic regions ≤ 51 nucleotides) functionally regulate protein-protein interactions in the brain and is altered in several neuronal diseases. However, little is known about the regulation and function of alternatively spliced microexons in striated muscle. Here, we investigated alternative splicing of a microexon in the synaptosome-associated protein 23 (*Snap23*) encoded gene. We found that inclusion of this microexon is developmentally regulated and tissue-specific, as it occurs exclusively in adult heart and skeletal muscle. The alternative region is highly conserved in mammalian species and encodes an in-frame sequence of 11 amino acids. Furthermore, we showed that alternative splicing of this microexon is mis-regulated in mouse models of heart and skeletal muscle diseases. We identified the RNA-binding proteins (RBPs) quaking (QKI) and RNA binding fox-1 homolog 2 (RBFOX2) as the primary splicing regulators of the Snap23 microexon. We found that QKI and RBFOX2 bind downstream of the Snap23 microexon to promote its inclusion, and this regulation can be escaped when the weak splice donor is mutated to the consensus 5’ splice site. Finally, we uncovered the interplay between QKI and muscleblind-like splicing regulator (MBNL) as an additional, but minor layer of Snap23 microexon splicing control. Our results are one of the few reports detailing microexon alternative splicing regulation during mammalian striated muscle development.

## GRAPHICAL ABSTRACT

**Figure:**
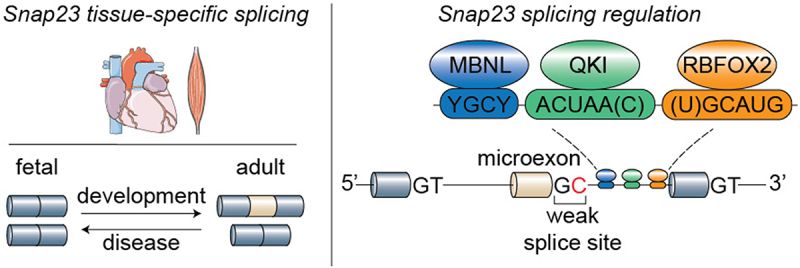

## Introduction

Alternative splicing is an RNA processing mechanism that enables a single gene to generate more than one mature RNA transcript. Approximately 95% of human multiexonic genes are alternatively spliced [1], thus considerably enhancing proteome diversity. Genome-wide studies in humans have revealed that each tissue in the body is characterized by a unique set of alternative splicing signatures [2,3], with brain, heart, and skeletal muscle exhibiting the most highly conserved splicing programmes [4]. Stage-specific splicing transitions often occur throughout organ maturation [5–8], and the aberrant expression of foetal splice isoforms in adult tissues is a hallmark of diseases such as autism spectrum disorder, cardiac hypertrophy, and myotonic dystrophy [5,9].

Variations in alternative splicing programmes observed between tissue types, developmental stages, or healthy versus diseased states are paralleled by differences in the expression levels of RNA-binding proteins (RBPs) [10]. RBPs are *trans*-acting factors that bind to *cis*-regulatory motifs located within a pre-mRNA transcript, and RBPs orchestrate splicing decisions by promoting or preventing spliceosome assembly at an alternative region. RBP-RNA interactions are governed by RBP expression levels as well as the position of motifs relative to the alternatively spliced region [11,12]. Additionally, different RBPs can bind to a target pre-mRNA transcript in a cooperative or competitive manner to control alternative splicing outcomes [11,12]. The myriad combinations of RBP-motif interactions, known as the splicing code, gives rise to tissue-specific and developmental stage-specific alternative splicing programmes [13–15].

In higher eukaryotes with large introns, the spliceosome uses the exon definition model for pre-mRNA processing and recognizes the 5’ and 3’ splice sites present in the introns surrounding an exon [16,17]. The optimal exon size for allowing the splicing machinery access to a transcript is approximately 51–300 nucleotides, and exons with lengths outside of this range are preferentially skipped [16,18–20]. Microexons are defined as exons that are smaller than 51 nucleotides, and are thus at a disadvantage for spliceosome recognition [18,21,22]. However, a growing number of studies are pointing to the importance of microexons for protein function and tissue development [22–24].

Global transcriptomic studies have revealed several key features of these ‘tiny but mighty’ [25] microexons. First, microexons are evolutionarily conserved across vertebrate species and exhibit tissue-specific splicing regulation, indicating a functional selective pressure at play [26,27]. Second, the length of these alternatively spliced microexons is often a multiple of three nucleotides, thereby maintaining the transcript open reading frame [22,23]. Third, microexons that exhibit tissue-specific alternative splicing regulation tend to map to disordered protein regions and have been found to remodel tissue-specific protein-protein interaction networks [28]. Lastly, microexons of functionally-related genes tend to be alternatively spliced in a coordinated manner by the action of one or more master RBPs [26,27]. As such, the inclusion of these regions hinges upon the presence of regulatory features surrounding the microexons [26]. These features highlight the importance of studying how the inclusion of microexons is regulated as well as the functional roles conferred on the proteins that contain them.

Recent work in neurons has identified networks of functionally-related genes that contain highly conserved microexons [26,27]. In one study, alternative splicing of this network has been shown to be coordinately regulated by one RBP, serine/arginine repetitive matrix 4 (SRRM4, also known as nSR100), during neurogenesis and inclusion of the microexons impacts protein-protein interaction potential [27]. Notably, neurons from individuals with autism spectrum disorder exhibited mis-regulated splicing of the microexon network and a corresponding reduction in SRRM4/nSR100 expression [27]. Another study demonstrated that a network of microexons in human brain tissue is antagonistically regulated by two RBPs, RNA binding fox-1 (RBFOX) and polypyrimidine tract binding protein 1 (PTBP1), and the inclusion of these microexons alters protein-protein interaction domains [26]. Aside from studies in the brain, little is known about the regulation and function of alternatively spliced microexons in other tissues. Early work in *Drosophila melanogaster* uncovered a muscle-specific microexon in the troponin T (*TnT*) gene that is developmentally regulated by alternative splicing [29]. A more recent report identified a microexon in the murine mitochondrial calcium uptake 1 (*Micu1*) gene, which is included specifically in skeletal muscle and impacts the MICU1 calcium binding capability [30]. Additional studies have identified microexons in the clathrin heavy chain (*Cltc*) [31] and bridging integrator 1 (*Bin1*) [32] genes that are differentially spliced in skeletal muscle. However, the regulatory mechanisms underlying the muscle-specific inclusion of these microexons have not been explored. In striated (heart and skeletal) muscle, numerous genes encoding proteins involved in aspects of membrane trafficking dynamics contain microexons that are alternatively spliced throughout development [33,34]. Yet, a vast lack of understanding about tissue-specific microexon splicing signatures in heart and skeletal muscle persists.

The synaptosome-associated protein 23 (SNAP23) is a well-known protein involved in membrane trafficking. SNAP23 is associated with the plasma membrane in the cell and interacts with other proteins, including vesicle-associated membrane proteins (VAMPs) and syntaxins, to mediate proper vesicle docking and fusion during exocytosis [35]. SNAP23 is necessary for development and viability, as Snap23-deficient mouse embryos die pre-implantation from a small and degenerating blastocyte that fails to expand [36]. Despite its critical function and ubiquitous expression [37], our understanding of SNAP23 has primarily been limited to its role in neurons and adipocytes [38,39]. Recently, we reported a role for SNAP23 in skeletal muscle cells and found that SNAP23-mediated secretion of the insulin-like growth factor 1 (IGF1) is necessary for skeletal muscle cell differentiation [40]. The Snap23 pre-mRNA transcript contains a single microexon that is alternatively spliced in striated muscle [34,41]; however, the regulation and function of this microexon has yet to be elucidated.

In this study, we investigate how alternative splicing of the Snap23 microexon is regulated. We establish that the Snap23 microexon sequence is highly conserved in mammals, inclusion of this region occurs exclusively during development of striated muscle tissues, and that this alternative splicing pattern is reverted in models of heart and skeletal muscle diseases. We show that quaking (QKI) and RNA binding fox-1 homolog 2 (RBFOX2) are the main regulators that promote inclusion of the Snap23 microexon by binding sequence-specific motifs downstream of the alternative region. The muscleblind-like splicing regulator (MBNL) proteins also play a minor role in Snap23 microexon splicing that is influenced by QKI function. Finally, editing the weak splice donor to a strong splice site is sufficient to achieve robust microexon inclusion. Collectively, these findings highlight a mechanism underlying the tight spatiotemporal inclusion of microexons in striated muscle.

## Materials and methods

### Mouse models

The FVB/N (Charles River Labs) and the C57BL/6 (Jackson Labs) mouse colonies were maintained in an accredited Division of Comparative Medicine (DCM) housing facility at The University of North Carolina at Chapel Hill with 12-hour light and dark cycles. Animals had access to food and water *ad libitum*. Upon tissue harvest, mice were euthanized by the isoflurane drop method followed by either decapitation (for neonates) or cervical dislocation (for adults) as a secondary physical method of euthanasia. Harvested tissues were immediately snap frozen in liquid nitrogen until further downstream processing. The university’s Institutional Animal Care and Use Committee (IACUC) has reviewed and approved all procedures for compliance.

### Human tissues

Total RNA samples from the following human tissues were commercially sourced: foetal heart (Cell Applications, #1F30–50; Agilent Technologies, #540165), adult heart (Amsbio, R1234129–50; Cell Applications, #1H30–50; TakaRa, #636532), foetal skeletal muscle (Cell Applications, #1F60–50; Agilent Technologies, #540181), adult skeletal muscle (Amsbio, R1234171–50; Cell Applications, #1H60–50; TakaRa, #636534).

### Phylogenetic analysis

SNAP23 protein sequences for representative mammalian species were downloaded in FASTA format from the Ensembl genome browser and imported into the PRALINE multiple sequence alignment programme [42]. Phylogenetic trees were calculated by TimeTree 5 [43] using the list of species analysed by PRALINE. Species silhouettes were obtained from PyloPic, under the Public Domain Mark 1.0 licence.

### Cell culture

C2C12 myoblasts (ATCC®, CRL-1772^™^) were maintained in an undifferentiated state by culturing the cells under 50% confluency in growth medium comprised of Dulbecco’s Modified Eagle’s Medium (DMEM) supplemented with 10% foetal bovine serum (FBS). Once myoblasts reached a confluency of > 80%, cells were washed with PBS (pH 7.4) (137 mm NaCl, 2.68 mm KCl, 9.55 mm Na_2_HPO_4_-7 H_2_O, 1.76 mm KH_2_PO_4_) and differentiated into myotubes by culturing them in differentiation medium comprised of DMEM supplemented with 2% horse serum (HS). Cells were incubated at 37°C in a 5% CO_2_ atmosphere.

### Delivery of small interfering RNAs (si-RNAs)

Undifferentiated C2C12 cells were plated in 6-well dishes at a density of 1.0 × 10^5^ cells per well in growth medium. All cells were transfected with 10 pmol/mL of each si-RNA (Supplementary Table S1) using the Lipofectamine RNAiMax Reagent (Invitrogen, #137778), including double and triple knockdowns experiments. For depletion of CUGBP Elav-like family member 1 (Celf1), Celf2, muscleblind-like splicing regulator 1 (Mbnl1), Mbnl2, embryonic lethal, abnormal vision-like 1 (Elavl1), and quaking (Qki): (a) approximately 24 hours after plating, cells were transfected, (b) the next day, cells were washed with PBS (pH 7.4) and differentiated into myotubes using differentiation medium, (c) after a total of four to five days of differentiation, myotubes were washed with PBS (pH 7.4) and processed for either RNA or protein extraction. For depletion of polypyrimidine tract binding protein 1 (Ptbp1) and Ptbp2: (a) approximately 24 hours after plating, cells were transfected, (b) between 24–48 hours later, cells were washed with PBS (pH 7.4) and processed for either RNA or protein extraction. For depletion of RNA binding fox-1 homolog 2 (Rbfox2): (a) approximately 24 hours after plating, cells were washed with PBS (pH 7.4) and differentiated into myotubes using differentiation medium, (b) after two days of differentiation, cells were transfected, (c) cells were differentiated for another 2 days, (d) myotubes were then washed with PBS (pH 7.4) and processed for either RNA or protein extraction.

### RNA extraction

RNA was extracted from both C2C12 cells and mouse tissues using TRIzol Reagent (Invitrogen, #15596018), unless otherwise specified. Tissues were first homogenized (6,500 rpm, 2 × 25 seconds) using a Precellys-24 homogenizer (Bertin Instruments) in lysing matrix D tubes (MP Biomedicals, #6913500) containing 1 mL of TRIzol Reagent and then placed on ice. Once cells and tissues were completely lysed in TRIzol Reagent, RNA extraction proceeded according to the manufacturer’s protocol. RNA concentrations were measured using a Nanodrop Lite spectrophotometer (Thermo Fisher Scientific, LT1497).

### Reverse transcription

RNA (1–2 µg) was reverse transcribed into cDNA using the High-Capacity cDNA Reverse Transcription Kit (Applied Biosystems, #4368813) according to the manufacturer’s protocol. The thermocycler for the reverse transcription reaction was programmed as follows: (i) 25°C for 10 minutes, (ii) 37°C for 120 minutes, (iii) 85°C for 5 minutes, (iv) 4°C pause.

### PCR evaluation of alternative splicing

Primers used for PCR assays were diluted to a final concentration of 0.5 µM in GoTaq Green Master Mix (Promega, M7123) and nuclease-free water. For evaluating endogenous Snap23 alternative splicing in mouse and human samples, the primers were designed to target the constitutive exons flanking the alternatively spliced microexon: Snap23–33-F_mouse (5’-AGA-AGA-AGG-CAT-GGA-CCA-AA-3’), Snap23–33-R_mouse (5’-AGT-TTG-CTG-AGG-CTG-ACC-AT-3’), Snap23–33-F_human (5’-CCT-TTG-TGT-CTG-CCC-ATG-TA-3’), Snap23–33-R_human (5’-TGC-AAG-GTG-AGT-TTT-CTC-CA-3’). The thermocycler for the PCR reaction was programmed as follows: (i) 95°C for 75 seconds, (ii) 27 cycles of 95°C for 45 seconds, 57°C for 45 seconds, 72°C for 1 minute, (iii) 72°C for 10 minutes, (iv) 25°C pause. For evaluating alternative splicing of the Snap23 minigenes, the RSV5U and TNIE4 primers were used as previously reported [44]. In this case, the thermocycler for the PCR reaction was programmed as follows: (i) 95°C for 1 minute, (ii) 23 cycles of 95°C for 1 minute, 55°C for 1 minute, 72°C for 30 seconds, (iii) 72°C for 5 minutes, (iv) 25°C pause.

PCR products were loaded alongside the pUC19 ladder (Thermo Fisher Scientific, SM0221) into a 6% polyacrylamide gel in TAE buffer (40 mm Tris, 20 mm acetic acid, 1 mm EDTA (pH 8.0)), and the bands were resolved at 140–150 V for 2.5–3 hours. The DNA was stained by submerging the gel into an aqueous solution of 0.4 µg/mL ethidium bromide (MP Biomedicals, ETBC1001) for 10 minutes, and the gel was imaged on the ChemiDoc XRS+ Imaging System (Bio-Rad). Alternative splicing was quantified by densitometry using the Image Lab software (Bio-Rad), and the percent spliced in (PSI) [3] was calculated.

### Quantitative real-time PCR (qPCR)

TaqMan probes (Supplementary Table S2) used for qPCR assays were diluted in TaqMan Fast Advanced Master Mix (Thermo Fisher Scientific, #4444557) and combined with 50–100 ng of cDNA in quadruplicate. The reactions were analysed using a Real-Time PCR machine (Applied Biosystems). The thermocycler for the qPCR reaction was programmed as follows: 40 cycles of (i) 50°C for 2 minutes, (ii) 95°C for 20 seconds, (iii) 95°C for 1 second, (iv) 60°C for 20 seconds. The RNA abundance of target genes was estimated relative to the expression level of a housekeeping gene. Fold changes in RNA expression were calculated by applying the delta-delta cycle threshold (ΔΔC_T_) quantification method.

### Protein extraction

Protein was extracted from C2C12 cells using RIPA buffer (50 mm Tris (pH 7.5), 150 mm NaCl, 5 mm EDTA (pH 8.0), 1% Triton X-100, 0.1% SDS, 0.5% sodium deoxycholate) supplemented with protease and phosphatase inhibitors (Millipore Sigma, PPC2020). Cells were placed on ice, washed with ice-cold PBS (pH 7.4), and lysed in ice-cold RIPA buffer (150 mm NaCl, 5 mm EDTA (pH 8.0), 50 mm Tris (pH 8.0), 1% Triton X-100, 0.5% sodium deoxycholate, 0.1% SDS, 1X protease and phosphatase inhibitors). Whole cell lysates were incubated on ice for 15 minutes, sonicated in an ice bath at 75 V for 3 minutes (30 seconds on, 30 seconds off), incubated again on ice for 15 minutes, and centrifuged at 18,000 × g for 10 minutes at 4°C. Protein was extracted from mouse tissues using HEPES/sucrose buffer (10 mm HEPES (pH 7.2), 320 mm sucrose, 1 mm EDTA (pH 8.0)) supplemented with protease and phosphatase inhibitors (Millipore Sigma, PPC2020). Tissues were weighed and then placed in lysing matrix D tubes (MP Biomedicals, #6913500) with HEPES/sucrose buffer to obtain a final concentration of approximately 5 mg/mL according to the following calculation: (mg tissue x 10% expected yield)/mL HEPES/sucrose buffer = mg/mL final concentration. Tissues were homogenized (6,500 rpm, 2 × 25 seconds) using a Precellys-24 homogenizer (Bertin Instruments). Lysates were centrifuged at 10,000 rpm for 3 minutes at 4°C then transferred to fresh tubes. SDS was added to a final concentration of 1%. Lysates were then rotated for 2 hours at 4°C, sonicated in an ice bath at 75 V for 3 minutes (30 seconds on, 30 seconds off), and centrifuged at 18,000 × g for 10 minutes at 4°C. Protein concentration was assessed using the Prometheus BCA Protein Assay kit (Genesee Scientific, #18–441).

### Western blot assays

Protein samples (15–35 µg) were combined with loading buffer (50 mm Tris-HCl (pH 6.8), 12.5 mm EDTA (pH 8.0), 10% glycerol, 2% SDS, 0.02% bromophenol blue, 360 mm beta-mercaptoethanol) and boiled at 95°C for 5 minutes. Denatured proteins were loaded alongside the All Blue (Bio-Rad, #161–0373), Unstained (Bio-Rad, #161–0396), or Kaleidoscope (Bio-Rad, #161–0375) ladders into either a 4–15% gradient Mini-Protean TGX Stain-Free gel (Bio-Rad, #456–8084) or a 12% long polyacrylamide gel (prepared in-house). Protein bands were resolved by electrophoresis in Running Buffer (25 mm Tris, 192 mm glycine, 3.5 mm SDS) at either 90 V for 90 minutes (mini gels) or 100 V for 60 minutes followed by 170 V for 3 hours (long gels). Proteins were then transferred by electrophoresis in Transfer Buffer (25 mm Tris, 192 mm glycine, 20% methanol) at 100 V for 60 minutes onto a 0.45 µm Immobilon-FL PVDF membrane (Millipore Sigma, IPFL85R). Total protein was visualized by either UV light exposure on the ChemiDoc XRS+ Imaging System (Bio-Rad) or stained for 10 minutes with Ponceau S solution (Sigma Aldrich, P7170). The membranes were blocked at room temperature for 60 minutes with 5% non-fat dried milk (RPI, M17200) in Tris-buffered saline (19 mm Tris (pH 7.6), 2.7 mm KCl, 137 mm NaCl) containing 0.1% Tween 20 (TBST). The membranes were then incubated at 4°C overnight with the respective primary antibodies diluted in a 1% bovine serum albumin (BSA) in TBS (BioWorld, #40220068) solution (Supplementary Table S3). The next day, the membranes were washed with TBST three times for 10 minutes each. The membranes were then incubated at room temperature for 90 minutes with either a goat anti-rabbit (Invitrogen, SA5–35571) or a goat anti-mouse (Invitrogen, SA5–35521) IgG (H+L) secondary antibody diluted 1:10,000 in a 1% BSA in TBS (BioWorld, #40220068) solution. The membranes were again washed with TBST three times for 10 minutes each and thenimaged on the Odyssey CLx Blot Images (Li-Cor). Bands were quantified by densitometry using the Image Studio software (Li-Cor). Protein levels were normalized to total transferred protein (full lanes) visualized by either the ChemiDoc XRS+ Imaging System (Bio-Rad) for the mini gels or the Ponceau S solution for the long gels.

### Left anterior descending coronary artery ligation (LAD)

Female FVB/N mice (approximately 12 weeks old) were weighed and prepared for surgery by removing the hair from the chest wall area. Animals were administered anaesthetic (100 mg/kg ketamine and 15 mg/kg xylazine) by intraperitoneal injection and also received an isoflurane/oxygen mixture by inhalation as needed. Mice were placed on their backs and positioned on an operating microscope with a heated base and electrocardiographic monitoring system. The paws were taped to the surface of the heating platform, and body temperature was maintained at approximately 37°C. Mice were endotracheally intubated through the mouth using a piece of PE-90 tubing that had one end bevelled at a 45° angle. Throughout the procedure, the mice were ventilated (tidal volume = 1.0 mL; rate = 120 breaths/minute) using a small animal respirator (Harvard Apparatus). The chest wall area was cleaned three times by applying isopropanol and povidone-iodine solutions and then covered with a sterile drape. First, an incision (1.5 cm) was made on the left chest landmarked between rib 3 and rib 4. Next, a transverse section of the chest muscles was made to expose the thoracic cage. Two 6–0 silk sutures were tied through the fourth intercostal space and placed around the upper and lower ribs. The ribs and thymus were gently displaced to fully expose the left aspect of the heart and the left main coronary artery system. At the bottom of the left anterior descending artery, a 7–0 monofilament polypropylene suture was tied through the myocardium and into the anterolateral left ventricular wall, closer to the apex of the heart [45]. Occlusion of the left coronary artery was confirmed by assessing myocardial blanching, which is indicative of disrupted coronary flow. The chest muscle incisions were closed with an absorbable suture. The skin incisions were closed with Vetbond and a 5–0 monofilament suture. Animals were gradually weaned from the respirator, and the endotracheal tube was removed once spontaneous breathing had resumed. Animals were placed under a heating lamp during recovery. Mice were administered analgesic (0.05–0.1 mg/kg buprenorphine) by subcutaneous injection. The first dose of analgesic was given at the same time as the anaesthetic. Subsequent doses of antibiotic and analgesic were given every 12 hours for a minimum of two days. One week after surgery, mice were euthanized. The infarct, border, and distal zones were each dissected from the ventricles. Tissues were placed in lysing matrix D tubes (MP Biomedicals, #6913500) with 1 mL of TRIzol Reagent (Invitrogen, #15596018) and snap frozen in liquid nitrogen.

### Transverse aortic constriction (TAC)

This procedure was performed as previously reported [46]. Briefly, male C57BL/6 mice (approximately 12–16 weeks old) were anaesthetised with 0.5–1.0 L/minute oxygen and 2% isoflurane. Animals were prepared for surgery by removing the hair from the chest wall area. Mice were placed on their backs and positioned on an operating microscope with a heated base to maintain body temperature at approximately 37°C. Mice were endotracheally intubated through the mouth using a piece of PE-90 tubing. Throughout the procedure, the mice were ventilated (tidal volume = 0.1–0.3 mL; rate = 125–150 breaths/minute) using a small animal respirator (Harvard Apparatus). The chest wall area was cleaned three times by applying isopropanol and then covered with a sterile drape. An incision was made on the left chest landmarked by rib 2. The sternum was retracted, then the thymus and fat tissue were separated from the aortic arch. One 6–0 silk suture was placed between the innominate and left carotid arteries. A 27.5 gauge blunt needle was placed parallel to the transverse aorta while two knots were quickly tied. For the control (sham) animals, no knots were tied. The rib cage and skin incisions were closed with 6–0 prolene sutures using interrupted and continuous suture patterns, respectively. Animals were gradually weaned from the respirator, and the endotracheal tube was removed once spontaneous breathing had resumed. Animals were placed under a heating lamp during recovery. Mice were administered analgesic (0.1 mg/kg buprenorphine) by intraperitoneal injection. One week after surgery, mice were anesthetised and body temperature was maintained at approximately 37°C. Doppler probes were placed on either side of the neck to assess the degree of pressure overload. Mice with a right carotid/left carotid flow velocity ratio of 5–10 were determined to have successful ligation and were included for further analysis. Seven weeks later, the mice were anesthetised again and body temperature was maintained at approximately 37°C. Echocardiography was performed using the VeVo 770 Imaging System (VisualSonics, Canada) [47] prior to euthanization. The entire ventricle was dissected. Tissues were placed in lysing matrix D tubes (MP Biomedicals, #6913500) with 1 mL of TRIzol Reagent (Invitrogen, #15596018) and snap frozen in liquid nitrogen.

### Barium chloride injection

Male and female FVB/N mice (approximately 130 days old) were anaesthetised with 2 L/minute oxygen and 4% isoflurane. Legs were sprayed with 70% ethanol. Using a U-40, 29-Gauge Insulin Syringe (UltiCare VetRx, A90409), each animal was injected with 30–50 µL of filter-sterilized 0.9% w/v saline solution (Thermo Fisher Scientific, S271–3) into the right *tibialis anterior* (TA) muscle and 30–50 µL of 1.2% w/v BaCl_2_ solution (50 mm) diluted in saline (Thermo Fisher Scientific, B34–100) into the left TA muscle. The TA muscles were harvested at 4, 14, and 41 days post BaCl_2_ injection. Tissues were placed in lysing matrix D tubes (MP Biomedicals, #6913500) with 1 mL of TRIzol Reagent (Invitrogen, #15596018) and snap frozen in liquid nitrogen.

### Molecular cloning of RBP constructs

The pGEX plasmid backbone was linearized by digestion with BamHI (NEB, R0136) and NotI (NEB, R0189) restriction enzymes. The linearized backbone was purified by running the digested product on an agarose gel and extracting the DNA using the QIAquick Gel Extraction Kit (Qiagen, #28704). The QKI sequence containing amino acids 11–213 (UniProt Accession: Q96PU8) and the RBFOX2 sequence containing amino acids 91–227 (UniProt Accession: O43251) were subcloned into the pGEX backbone with primers containing pGEX overlaps (5’-GTC-AGC-GTG-AAC-CGG-GAT-CC-3’) and (5’-CGC-CGG-CGT-AGC-ACT-GAC-TGA-C-3’), tagging QKI and RBFOX2 to GST (glutathione S-transferase) and SBP (streptavidin-binding peptide). To generate the purified plasmids, Stellar competent cells (Takara Bio, #636763) were transformed with either the QKI or RBFOX2 vector clones. The GST-SBP-QKI and GST-SBP-RBFOX2 plasmids were cultured overnight at 37°C in Luria-Bertani (LB) broth supplemented with 100 µg/mL ampicillin. DNA was isolated from grown cultures using the QIAprep Spin Miniprep Kit (Qiagen, #27104).

### Bacterial expression and protein purification of RBP constructs

Rosetta competent cells (Millipore Sigma, #70954) were transformed with either the GST-SBP-QKI or GST-SBP-RBFOX2 clones. The transformed cells were cultured at 37°C in LB broth supplemented with 100 µg/mL ampicillin and 25 µg/mL chloramphenicol until an optical density of approximately 0.6 was reached. Cultures were brought to 16°C and induced with 0.5 mm isopropyl β-d-1-thiogalactopyranoside overnight. Cells were then harvested and lysed with a buffer containing 20 mm HEPES, 200 mm NaCl, 1% Triton X-100, 4 mm MgCl2, 5 mm DTT, 2.5 mm phenylmethylsulfonyl fluoride, and protease inhibitors (Thermo Fisher Scientific, A32955). The protein lysates were sonicated at 100% amplitude for a total of 64 seconds using a Q125 sonicator (Qsonica). After sonication, 500 units of benzonase and 3 units of RQ1 per litre of culture were added, and the lysates were rotated at room temperature for 20 minutes. Lysates were centrifuged at 37 krcf for 35 minutes at 4°C. GST-tagged proteins were captured on 5 mL GSTrap HP column (Cytivia, #17528202) on a ÄTKA Pure HPLC. Columns were washed using Wash Buffer (200 mm NaCl, 20 mm HEPES, 0.01% Triton X-100) and eluted using Elution Buffer (20 mm GSH, 50 mm Tris Base (pH 8.0)). Protein was concentrated using a 10 kDa spin filter (Cytiva, #28932296) and measured using the Pierce BCA Assay Kit (Thermo Fisher Scientific, #22660). Purity was assessed by running the protein on a polyacrylamide gel and staining with Coomassie blue. Proteins were loaded alongside the Pre-stained Protein Ladder (Thermo Fisher Scientific, #26619).

### Fluorescence polarization assays

Short RNA oligonucleotides (19–20 nt) that spanned the putative binding motifs for QKI (QKI #1 and QKI #2) and RBFOX2 (RBFOX2 #1 and RBFOX2 #2) were designed and synthesized to contain a 6-FAM (fluorescein) label (Supplementary Table S4). Two independent preparations of purified GST-SBP-QKI and GST-SBP-RBFOX2 proteins were serially diluted in binding buffer (100 mm NaCl, 20 mm HEPES, 0.01% Triton, 5 mm DTT) containing 0.2 µL/mL BSA and incubated with 5 nM of the respective RNA oligonucleotide. Fluorescence polarization was measured two independent times for each protein preparation (4 assays total) using a PHERAstar Microplate Reader (BMG Labtech). For RBFOX2 #2, fluorescence polarization was measured after two hours of incubation with RNA oligonucleotide. Each of the 4 assays was performed in technical duplicates. These technical duplicates were averaged. The data were plotted and fitted to a 4-parameter logistical binding model using Prism.

### Construction of minigene and mutants

The RHCglo minigene backbone was a gift from Dr. Thomas Cooper (Addgene plasmid #80169; http://n2t.net/addgene:80169; RRID:Addgene_80169). RHCglo plasmid DNA was linearized by digestion with SalI (NEB, R3138) and SpeI (NEB, R3133) restriction enzymes and then incubated with calf intestinal phosphatase (CIP) (NEB, M0525) to prevent plasmid re-ligation. The linearized backbone was purified by running the digested product on a 1% agarose gel and extracting the DNA using the NucleoSpin Gel and PCR Clean-Up Kit (Macherey-Nagel, #740609). The Snap23 minigene insert was amplified by PCR from DNA isolated from C2C12 cells using Q5 polymerase (NEB, M0492) and primers containing the SalI and SpeI restriction sites (Supplementary Table S5). The region of interest to be inserted included the Snap23 microexon along with 300 base pairs of the upstream and 300 base pairs of the downstream introns. Amplified product was purified using the NucleoSpin Gel and PCR Clean-Up Kit (Macherey-Nagel, #740609) and then digested with SalI (NEB, R3138) and SpeI (NEB, R3133) restriction enzymes. The RHCglo backbone and Snap23 minigene insert were ligated using the Quick Ligation Kit (NEB, M2200) generating the Snap23-ex33nts wild-type plasmid. DH5α competent cells (Thermo Fisher Scientific, #18265017) were then transformed with the Snap23-ex33nts wild-type plasmid. RBP motif deletions were made using the Q5 Site-Directed Mutagenesis Kit (NEB, E0552S). Mutant-specific primers were used in combination with different plasmid backbones to achieve the desired edits (Supplementary Table S5). In this manner, the following mutant minigene plasmids were generated: Snap23-ex33nts ΔQKI core #1, Snap23-ex33nts ΔQKI core #2, Snap23-ex33nts ΔQKI core #1 + 2, Snap23-ex33nts ΔRBFOX2 #1, Snap23-ex33nts ΔRBFOX2 #1 + 2, Snap23-ex33nts ΔQKI core #1 + 2 and ΔRBFOX2 #1 + 2, Snap23-ex33nts sub-SpliceDonor-CtoT (Supplementary Table S6). All plasmids generated were cultured overnight at 37°C in LB broth (Thermo Fisher Scientific, BP1426) supplemented with 100 µg/mL ampicillin (Sigma Aldrich, A5354). DNA was isolated from grown cultures using the ZymoPure II Plasmid Maxiprep Kit (Zymo Research, D4203). Constructs were verified by Sanger sequencing using RHCglo-specific primers (Supplementary Table S5).

### Delivery of wild-type and mutant minigene plasmids

Undifferentiated C2C12 cells were plated in 6-well dishes at a density of 1.2 × 10^5^ cells per well in growth medium. The next day, cells were transfected with 2.5 µg of each plasmid using the Lipofectamine 3000 Reagent (Thermo Fisher Scientific, L3000001). For the cells that were also depleted of MBNL1 + MBNL2, the transfection of the minigene plasmids occurred on the same day as the transfection of the si-RNAs. Two days later, cells were washed with PBS (pH 7.4) and cultured in differentiation medium. After three to four days of differentiation, the cells were washed with PBS (pH 7.4) and pelleted by centrifugation at 300 × g for 5 minutes. RNA was harvested from the pellets using the RNeasy Mini Kit (Qiagen, #74104) according to the manufacturer’s protocol.

### Morpholino delivery

Morpholino antisense oligonucleotides (Gene Tools) (Supplementary Table S7) were resuspended in dH_2_O for a stock concentration of 1 mm. The Neon transfection system (Invitrogen, MPK5000) was used for electroporation delivery of the morpholinos into C2C12 cells. Undifferentiated C2C12 cells were spun at 400 × g for 5 minutes. The pellet was resuspended in PBS (pH 7.4) to wash the cells and then spun again at 400 × g for 5 minutes. The cells were resuspended at a final concentration of 6.25 × 10^6^ cells/mL in Resuspension Buffer R (Invitrogen, MPK10025). Each morpholino was delivered into the cells by electroporation at a final concentration of 10 µM using the 100 µL Neon tip at the following settings: 1,650 V pulse voltage, 10 millisecond pulse width, 3 pulses. Cells were plated in 6-well dishes containing growth medium. The next day, cells were washed with PBS (pH 7.4) and cultured in differentiation medium. After four days of differentiation, the cells were washed with PBS (pH 7.4) and RNA was harvested as described above.

### Statistical analysis

Statistical analysis was performed using Excel (Microsoft) or Prism (GraphPad Software Inc). The statistical tests used in each experiment are detailed in the figure legends. Data were considered statistically significant when *p* ≤ 0.05, and all data are reported as the mean ± the standard error of the mean (SEM).

## Results

### Alternative splicing of the Snap23 microexon is developmentally regulated in striated muscle tissues

**Figure 1.**
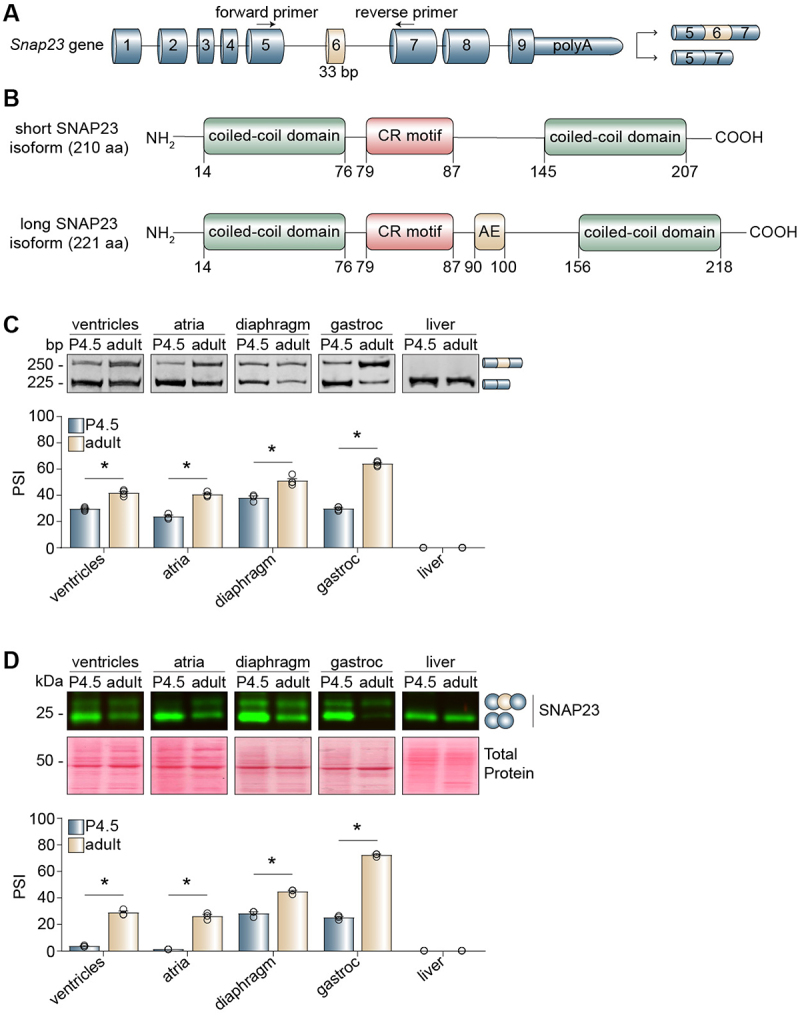
Alternative splicing of the Snap23 microexon is developmentally regulated in striated muscle tissues. (A) Schematic of the murine *Snap23* gene locus. PCR primers (arrows) bind the constitutive exons (blue) flanking the alternative microexon (exon 6, gold). (B) Schematic of the two SNAP23 protein isoforms produced by alternative splicing of the microexon. Protein domains were identified using PROSITE (expasy) and UniProt. (C-D) Different mouse tissues at postnatal day 4.5 (P4.5) and adulthood (3–4 months old) were evaluated by reverse transcription PCR (RT-PCR) (C) and western blot assays (D). The percent spliced-in (PSI) values were calculated by densitometry. Results are shown as the mean ± SEM, *p ≤ 0.05 versus P4.5, unpaired t-test with Welch’s correction, *n* = 3–4 independent replicates. aa: amino acids; AE: alternative exon; bp: base pairs; CR: cysteine-rich; gastroc: gastrocnemius.

In mice, the *Snap23* gene contains nine exons. Only exon 6 (Figure 1A, gold cylinder) is alternatively spliced, during postnatal development of mouse heart [34] and skeletal muscle [41]. Given its small size (33 base pairs), this alternative region is considered a microexon [22]. This microexon encodes an in-frame peptide of 11 amino acids (FSVGDCFFETR) that is positioned between the two coiled-coil domains and located downstream of the cysteine-rich motif (Figure 1B). We first asked whether alternative splicing of the Snap23 pre-mRNA is ubiquitous or tissue-specific. We thus assessed Snap23 microexon inclusion across a broad panel of mouse tissues. We performed reverse transcription-PCR (RT-PCR) assays during postnatal development using primers that bind the constitutive exons (exons 5 and 7) flanking the alternatively spliced microexon (Figure 1A). The PCR products were separated by polyacrylamide gel electrophoresis, and the percent spliced in (PSI) [3] was quantified by densitometry. We observed that inclusion of the Snap23 microexon is restricted to the development of heart and skeletal (striated) muscle, as it was not included in any of the other tested tissues (Figure 1C and Figure S1A). Skeletal muscle tissues can be further categorized by the fibre types they contain: fast-twitch or slow-twitch [48]. The two fibre types are distinguished by structural and metabolic differences that influence muscle contractility [49], and genes encoding structural proteins have been shown to exhibit fibre type-specific alternative splicing [50–52]. We thus evaluated Snap23 microexon splicing in fast-twitch and slow-twitch skeletal muscles and found that this microexon is included in both types of muscle (Figure S1B). Furthermore, western blot assays confirmed that the alternative splicing switch is consistent at the protein level (Figure 1D), despite changes in total SNAP23 protein abundance throughout development (Figure S1C).

Knowing that the sequence and inclusion of microexons is often evolutionarily conserved in vertebrates [26,27], we examined Snap23 splicing in human striated muscles. Indeed, we found that the developmentally regulated splicing transition occurs in human heart and skeletal muscle (Figure S2A). Phylogenetic analysis of the protein peptide sequence encoded by the SNAP23 microexon revealed that this region is highly conserved throughout evolution in mammals (Figure S2B). These data demonstrate that alternative splicing of the Snap23 microexon is evolutionarily conserved in a tissue- and developmental stage-specific manner.

### The Snap23 microexon is mis-spliced in mouse models of heart and skeletal muscle diseases

**Figure 2.**
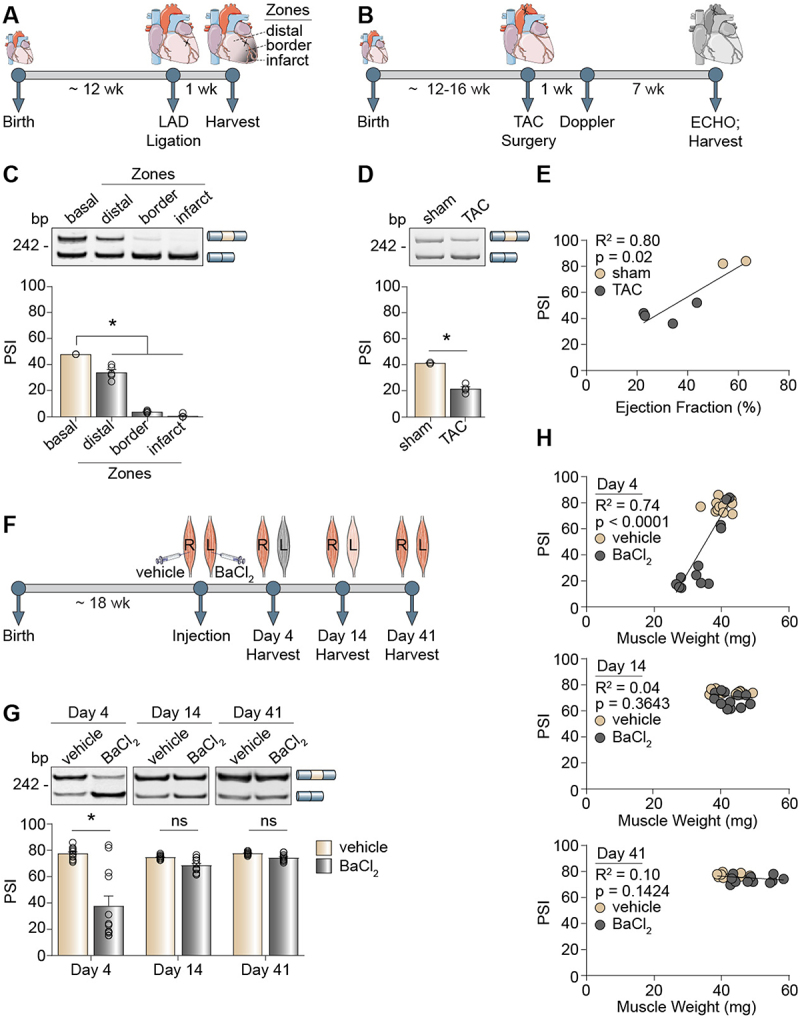
The Snap23 microexon is mis-spliced in mouse models of heart and skeletal muscle diseases. (A-B) Timeline of experiments using left anterior descending (LAD) ligation (A) and transverse aortic constriction (TAC) (B) surgeries in mice. (C-D) Alternative splicing of the Snap23 microexon in LAD (C) and TAC (D) models of heart disease was evaluated by RT-PCR assays. (E) Scatterplot correlating microexon inclusion (percent spliced-in, PSI) with cardiac ejection fraction after TAC surgery. (F) Timeline of the barium chloride (BaCl_2_) injection performed in mice. Red = healthy muscle, pink = regenerating muscle, gray = injured muscle. (G) Alternative splicing of the Snap23 microexon 4, 14 and 41 days after BaCl_2_ injection was evaluated by RT-PCR assays. (H) Scatterplots correlating inclusion of the Snap23 microexon (PSI) with skeletal muscle weights after BaCl_2_ injection. PSI values were calculated by densitometry. Results are shown as the mean ± SEM, *p ≤ 0.05 versus the respective control condition (basal, sham, vehicle), ordinary one-way ANOVA with Tukey’s multiple comparisons test, *n* = 3–4 independent replicates for LAD (A), unpaired t-test with Welch’s correction, *n* = 2–4 independent replicates for TAC (B), two-way ANOVA with Tukey’s multiple comparisons test and simple linear regression analysis, *n* = 11–12 independent replicates for BaCl_2_ (G-H). bp: base pairs; ECHO: echocardiogram; ns: not significant; wk: week(s).

A hallmark of heart and skeletal muscle pathologies is a reprogramming of alternative splicing to foetal patterns in adult tissues [53–57]. We thus assessed whether the Snap23 microexon is aberrantly spliced in diseased striated muscle tissues. We performed left anterior descending (LAD) ligation (Figure 2A) and transverse aortic constriction (TAC) surgeries (Figure 2B) on adult mice as models of myocardial infarction and pressure overload-induced heart failure, respectively. Following LAD ligation, we observed almost complete skipping of the Snap23 microexon that was strongest in the infarct area (99% reduction of microexon inclusion) and radiated out to the border zone (92% reduction of microexon inclusion), while in the distal region the reduction was less pronounced but still significant (29% reduction of microexon inclusion) (Figure 2C). Similarly, hearts exhibited a significant decrease in the inclusion of the Snap23 microexon after TAC surgery (Figure 2D), and the PSI strongly correlated with the ejection fraction, which is a measurement of how efficiently the heart is pumping blood (Figure 2E). The largest PSI values (high microexon inclusion) were found in healthy hearts indicated by high ejection fractions, and the smallest PSI values (low microexon inclusion) were detected in failing hearts indicated by low ejection fractions (Figure 2E, R^2^ = 0.80).

We then performed barium chloride (BaCl_2_) injection in the *tibialis anterior* (TA) muscle of adult mice as a model of skeletal muscle injury (Figure 2F) [58–60]. The TA muscle was injected locally with either 0.9% w/v vehicle saline solution (right leg) or 1.2% w/v BaCl_2_ solution (left leg) to induce muscle damage (Figure 2F). BaCl_2_ blocks potassium channels in the myofibers, triggering a series of events involving calcium overload, depolarization of the sarcolemma, proteolysis, and membrane rupture, which ultimately leads to necrosis [59]. Following this acute injury, quiescent muscle stem cells respond by re-entering the cell cycle and differentiating into myoblasts that can fuse with the damaged tissue for myofiber repair [60]. Indeed, BaCl_2_-induced muscle injury caused an acute reversion in splicing of the Snap23 microexon from inclusion to skipping, which was gradually restored as the tissue regenerated over time (Figure 2G). At day 4 post-injection, the PSI strongly correlated with muscle weight (Figure 2H, Day 4). As the tissue regenerated, both splicing and muscle weight were restored (Figure 2H, Day 14 and Day 41). We evaluated the mRNA expression of myogenic markers as an orthogonal approach to validate muscle injury and repair [58,61]. We observed elevated levels of the satellite cell activation marker, myogenic factor 5 (Myf5), along with the early proliferation markers, myogenic differentiation 1 (Myod1) and myogenin (Myog), in the BaCl_2_-treated tissues during muscle degeneration (Figure S3A-C, Day 4). Conversely, the late differentiation markers, myosin, heavy chain 1 (Myh1) and Myh2, were significantly downregulated during muscle degeneration (Day 4) and then recovered during muscle regeneration (Figure S3D-E, Day 14 and Day 41).

In summary, alternative splicing of the Snap23 microexon is dysregulated in mouse models of heart and skeletal muscle diseases, and skeletal muscle regeneration drives the observed splicing changes back towards microexon inclusion.

### The Snap23 developmental splicing transition is recapitulated during C2C12 cell differentiation

**Figure 3.**
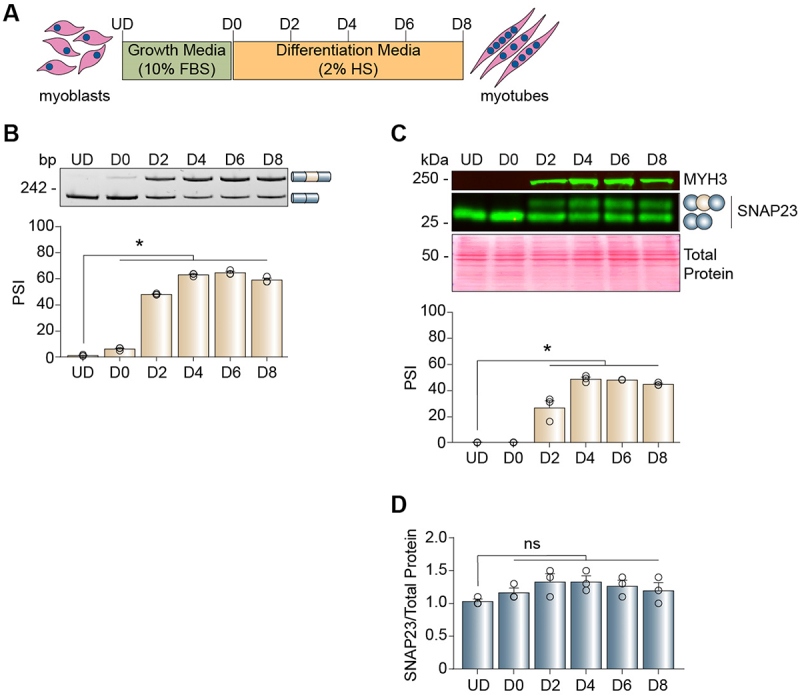
The Snap23 developmental splicing transition is recapitulated during C2C12 cell differentiation. (A) Schematic of C2C12 myoblast differentiation into myotubes. (B-C) Alternative splicing of the Snap23 microexon during C2C12 cell differentiation was evaluated by RT-PCR (B) and western blot (C) assays. The percent spliced-in (PSI) values were calculated by densitometry. (D) Total SNAP23 protein levels were evaluated by western blot assays and quantified by densitometry. Results are shown as the mean ± SEM, *p ≤ 0.05 versus UD, ordinary one-way ANOVA with Dunnett’s multiple comparisons test, *n* = 3 independent replicates. bp: base paris; D: differentiation day; FBS: foetal bovine serum; HS: horse serum; MYH3: myosin heavy chain 3; ns: not significant, UD: undifferentiated.

Skeletal muscle development starts with cell differentiation [62], and the C2C12 mouse myoblast cell line is a well-established model system for studying this early stage of muscle maturation [63,64]. In brief, mononucleated C2C12 myoblasts grown in enriched medium can be induced to fuse and differentiate into multinucleated myotubes by reducing the serum concentration in the medium (Figure 3A). We assessed alternative splicing of the Snap23 microexon throughout C2C12 cell differentiation by collecting RNA and protein at six specific time points (Figure 3A). We observed that the Snap23 microexon was completely skipped in undifferentiated myoblasts and was gradually included during their differentiation into myotubes (Figure 3B-C). Importantly, total SNAP23 protein levels did not change throughout differentiation (Figure 3D). This splicing pattern recapitulates what occurs *in vivo* in striated muscle (Figure 1C-D), establishing C2C12 cells as a useful model for studying the regulation of this tissue-specific microexon.

### Strength of the splice donor site determines the level of Snap23 microexon inclusion

**Figure 4.**
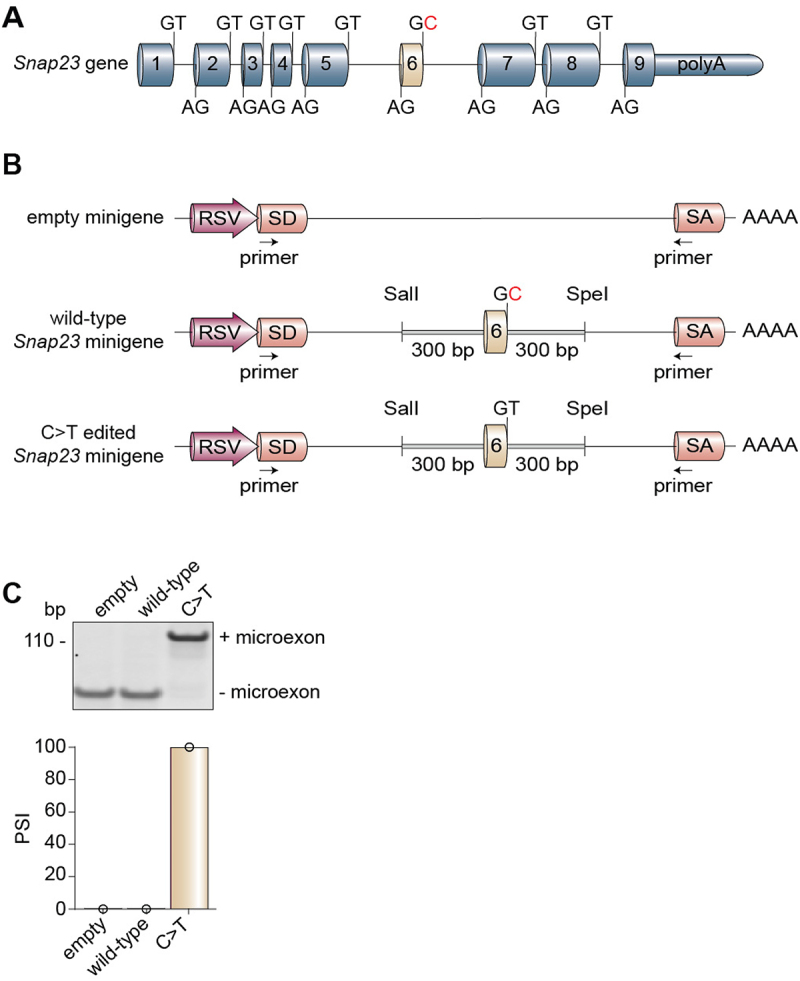
Strength of the splice donor site determines the level of Snap23 microexon inclusion. (A) Schematic of the murine *Snap23* gene locus annotated with the splice donor site and splice acceptor site sequences. (B) Schematics of the empty, wild-type, and edited Snap23 minigenes expressed in undifferentiated C2C12 cells. Arrows indicate the location of the primers used to evaluate alternative splicing of the Snap23 microexon in the minigenes. (C) Alternative splicing of the three minigene constructs in myoblasts was evaluated by RT-PCR assays. The percent spliced-in (PSI) values were calculated by densitometry. bp: base pairs; RSV: Rous sarcoma virus; SA: splice acceptor; SD: splice donor.

Recognition of the 5’ splice site by the spliceosome is the first step of the splicing reaction. The degree to which this splice site at the exon-intron junction is conserved can distinguish between a constitutive and alternative exon, where, in general, high conservation (strong splice site) coincides with constitutive splicing and low conservation (weak splice site) coincides with alternative splicing [65]. We noticed a weak splice site at the downstream exon-intron boundary of the Snap23 microexon (Figure 4A), leading us to ask whether introducing a C-to-T mutation is sufficient to achieve constitutive microexon inclusion. We therefore cloned the Snap23 microexon, along with 300 base pairs of the upstream and 300 base pairs of the downstream flanking introns, into the RHCglo minigene backbone [44] (Figure 4B). We assessed Snap23 splicing by RT-PCR assays using primers that exclusively bind to the minigene backbone and not the endogenous Snap23 mRNA. Indeed, we observed that the C-to-T change, converting the weak splice donor site (GC) into a strong splice donor site (GT), resulted in complete inclusion of the Snap23 microexon in undifferentiated C2C12 cells (Figure 4C). These data demonstrate that Snap23 microexon inclusion can be uncoupled from its temporal regulation upon editing the 5’ splice site and changing it to a strong donor sequence.

### MBNL, QKI, and RBFOX2 are positive splicing regulators of the Snap23 microexon

**Figure 5.**
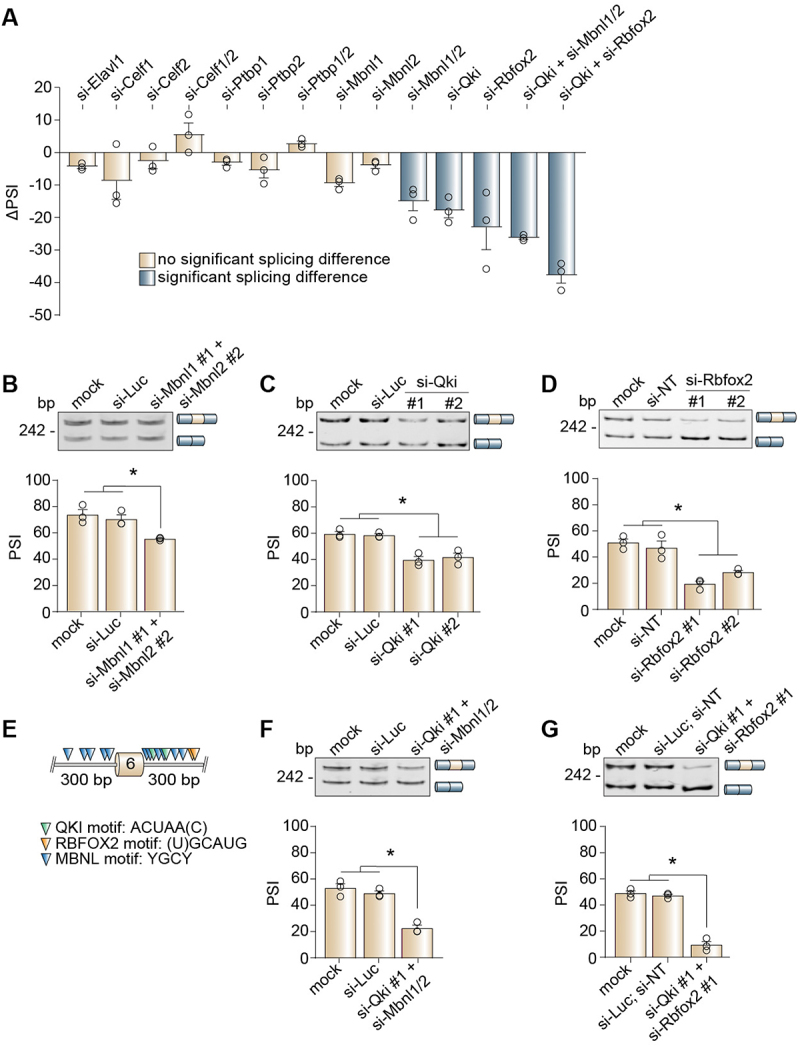
MBNL, QKI, and RBFOX2 are positive splicing regulators of the Snap23 microexon. (A) Differences in inclusion of the Snap23 microexon (ΔPSI) were plotted for each RBP knockdown. RNA was harvested from either undifferentiated myoblasts (si-Ptbp1, si-Ptbp2, si-Ptbp1 + si-Ptbp2) or differentiated myotubes (si-Elavl1, si-Celf1, si-Celf2, si-Celf1 + si-Celf2, si-Mbnl1, si-Mbnl2, si-Mbnl1 + si-Mbnl2, si-Qki, si-Rbfox2, si-Qki + si-Mbnl1 + si-Mbnl2, si-Qki + si-Rbfox2). The ΔPSI values were calculated as follows: PSI (si-RBP) – PSI (si-control). (B-D) Alternative splicing of the Snap23 microexon following depletion of MBNL1 + MBNL2 (B), QKI (C), or RBFOX2 (D) was evaluated by RT-PCR assays. The percent spliced-in (PSI) values were calculated by densitometry. (E) Schematic of the putative MBNL, QKI, and RBFOX2 binding motifs identified in the upstream and downstream intronic regions flanking the alternatively spliced Snap23 microexon. (F-G) Alternative splicing of the Snap23 microexon following depletion of QKI + MBNL1 + MBNL2 (F) or QKI + RBFOX2 (G) was evaluated by RT-PCR assays. PSI values were calculated by densitometry. Results are shown as the mean ± SEM, *p ≤ 0.05 versus mock and si-control (si-Luc or si-NT), ordinary one-way ANOVA with Tukey’s multiple comparisons test, *n* = 3 independent replicates. bp: base pairs; Luc: Luciferase; NT: non-targeting.

We hypothesized that recognition of the weak splice site has to be promoted by the action of specific splicing regulators. To identify the molecular players controlling the tissue-specific splicing of the Snap23 microexon, we selected a panel of RBPs that are known to regulate alternative splicing [66] and are expressed in striated muscle tissues as well as in C2C12 cells [33]: embryonic lethal, abnormal vision-like 1 (ELAVL1), CUGBP Elav-like family member 1 (CELF1), CELF2, polypyrimidine tract binding protein 1 (PTBP1), PTBP2, MBNL1, MBNL2, QKI, and RBFOX2. We depleted these RBPs in C2C12 cells using two independent small interfering RNAs (si-RNAs) per RBP (#1 and #2) and then assessed alternative splicing of the Snap23 microexon by RT-PCR assays in either undifferentiated myoblasts or differentiated myotubes, depending on the stage when each RBP was most highly expressed (Figure S4–5). We observed very small changes in Snap23 microexon inclusion after ELAVL1, CELF1, CELF2, CELF1 and CELF2, PTBP1, PTBP2, PTBP1 and PTBP2, MBNL1, or MBNL2 depletion (Figure 5A and Figure S6). However, the MBNL1 and MBNL2 double knockdown exhibited a more pronounced difference in the inclusion level relative to the single knockdowns (Figure 5A-B), consistent with reports of paralog compensation between the MBNL proteins in C2C12 cells [67]. Of the single RBP knockdowns, depletion of either QKI or RBFOX2 caused the most significant reductions in Snap23 microexon inclusion (Figure 5A,C-D).

Numerous RBPs can function both as negative and positive splicing regulators, depending on their binding position relative to the exon being spliced [11,68]. MBNL, QKI, and RBFOX2 are known to exhibit positional effects on the regulation of alternative splicing, acting as negative regulators to promote skipping when they bind to the upstream intron and acting as positive regulators to promote inclusion when they bind to the downstream intron [69–75]. Given that depletion of MBNL1 and MBNL2, QKI, or RBFOX2 led to increased skipping of the Snap23 microexon, we hypothesized that these RBPs function as positive regulators and predicted the presence of their respective binding motifs downstream of the microexon. MBNL, QKI, and RBFOX2 bind to well-defined consensus motifs: YGCY [66,74,76,77], ACUAA(C) [78,79] and (U)GCAUG [66,76,77], respectively. We thus searched within a 300 base pair window of the upstream and downstream intronic regions most proximal to the Snap23 microexon for putative motifs. Indeed, we found both the upstream and downstream intronic regions to be replete with the highly-conserved MBNL motif (Figure 5E, blue triangles). We further found two QKI motifs and two RBFOX2 motifs within the 300 base pair downstream intronic region (Figure 5E, green triangles and orange triangles, respectively) and none within the 300 base pair upstream intronic region.

Since we noticed that numerous MBNL motifs were proximal to the QKI motifs, we were curious whether there was some type of regulatory interplay between the MBNL proteins and QKI. Combined depletion of QKI, MBNL1, and MBNL2 resulted in a greater change in Snap23 microexon inclusion than what was observed with the individual knockdowns (Figure 5F). We also performed a double knockdown of QKI and RBFOX2 and found this combination led to the strongest effect on Snap23 microexon inclusion (Figure 5G).

In summary, during C2C12 cell differentiation, MBNL proteins, QKI, and RBFOX2 might promote Snap23 microexon inclusion through binding to motifs located downstream. Our results indicate that QKI and RBFOX2 are the primary regulators of Snap23 microexon splicing and that the MBNL proteins play an additional, but less pronounced regulatory role.

### QKI and RBFOX2 bind their putative motifs with high affinity in vitro

**Figure 6.**
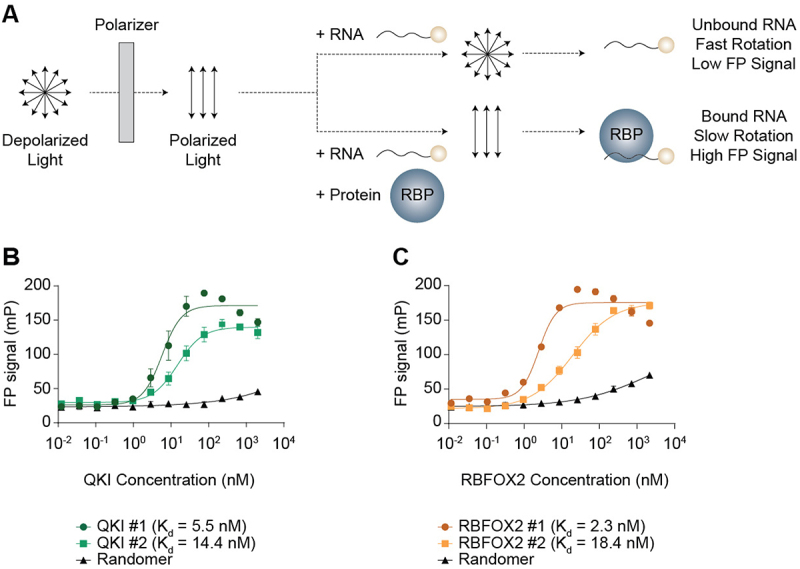
QKI and RBFOX2 bind their putative motifs with high affinity *in vitro*. (A) Schematic of the fluorescence polarization (FP) assay. (B-C) Binding curves for Snap23 oligonucleotides incubated with purified QKI (B) or RBFOX2 (C) proteins. Results are shown as mean ± SEM, nonlinear regression (curve fit), 4-parameter logistical binding model with least squares fit, *n* = 4 independent replicates.

We next probed whether the putative sites that we identified were indeed physically bound by the RBPs using *in vitro* fluorescence polarization assays, focusing on the two strongest regulators: QKI and RBFOX2. Short RNA oligonucleotides (19–20 nt) that spanned the putative binding motifs for QKI (QKI #1 and QKI #2) and RBFOX2 (RBFOX2 #1 and RBFOX2 #2) were synthesized and incubated with various concentrations of purified QKI or RBFOX2 proteins (Figure 6A). We observed high affinity binding for both the QKI #1 (K_d_ = 5.5 nM) and QKI #2 (K_d_ = 14.4 nM) oligonucleotides, whereas the randomer control sequence exhibited minimal binding (Figure 6B, compare green curves to black curve). Similarly, RBFOX2 bound strongly to the RBFOX2 #1 oligonucleotide *in vitro* (Figure 6C, K_d_ = 2.3 nM). Based on *in silico* folding predictions, we hypothesized that the RBFOX2 #2 oligonucleotide formed intermolecular secondary structures. Therefore, the reaction reached equilibrium after proceeding for 2 hours and enhanced the RNA-RBP interaction (Figure 6C, K_d_ = 18.4 nM). These data demonstrated that the candidate QKI and RBFOX2 motifs that we identified were inde bound by these RBPs *in vitro*, leading us to explore their occupancy and functional roles in culture using C2C12 cells.

### QKI and RBFOX2 motifs are necessary for alternative splicing regulation of the Snap23 microexon in muscle cells

**Figure 7.**
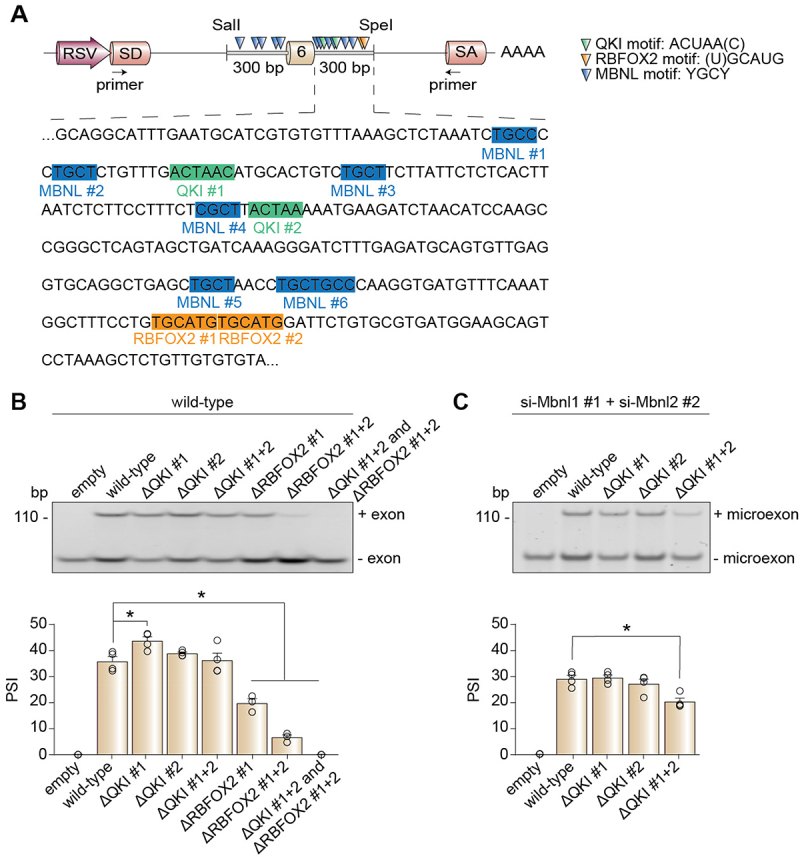
Putative QKI and RBFOX2 motifs are necessary for Snap23 microexon alternative splicing regulation. (A) Schematic of the wild-type Snap23 minigene expressed in C2C12 cells. (B) Alternative splicing of the Snap23 minigene in wild-type myotubes (differentiated for 4 days) after deletion of the putative RBP motifs was evaluated by RT-PCR assays. (C) Alternative splicing of the Snap23 minigene in myotubes depleted of MBNL1 + MBNL2 (differentiated for 4 days) after deletion of the putative RBP motifs was evaluated by RT-PCR assays. The percent spliced-in (PSI) values were calculated by densitometry. Results are shown as the mean ± SEM, *p ≤ 0.05 versus wild-type, one-way ANOVA with Dunnett’s multiple comparisons test, *n* = 3–4 independent replicates. bp: base pairs; RSV: Rous sarcoma virus; SA: splice acceptor; SD: splice donor.

We hypothesized that deletion of the QKI and RBFOX2 binding motifs would result in a splicing phenotype comparable to the RBP knockdown experiments (Figure 5). Using our wild-type Snap23 minigene construct, we deleted each of the identified QKI or RBFOX2 motifs (Figure 7A) and expressed the mutants in C2C12 cells. In differentiated C2C12 myotubes, the wild-type minigene construct was alternatively spliced as expected (Figure 7B, lane 2). Deletion of any combination of the QKI motifs did not affect splicing of the Snap23 microexon (Figure 7B, lanes 3–5); however, deletion of one or both of the RBFOX2 motifs resulted in a significant decrease in inclusion of the Snap23 microexon (Figure 7B, lanes 6–7), and removal of all four RBP motifs led to complete microexon skipping (Figure 7B, lane 8). These data indicated that QKI and RBFOX2 promote the inclusion of the Snap23 microexon by making the weak 5’ splice site more favourable for spliceosome recognition.

Given that we uncovered a potential regulatory interplay between the MBNL proteins and QKI (Figure 5F), we decided to express the Snap23 minigenes lacking the QKI motifs in C2C12 cells where we knocked down MBNL1 and MBNL2 (Figure S7). While there was no change in Snap23 microexon splicing upon single QKI motif deletions, we observed a significant reduction in Snap23 microexon inclusion when removal of both of the QKI motifs was paired with the MBNL1 and MBNL2 double knockdown (Figure 7C, lane 5), a phenotype not seen when this minigene was expressed in wild-type C2C12 cells expressing MBNL1 and MBNL2 (Figure 7B, lane 5).

**Figure 8.**
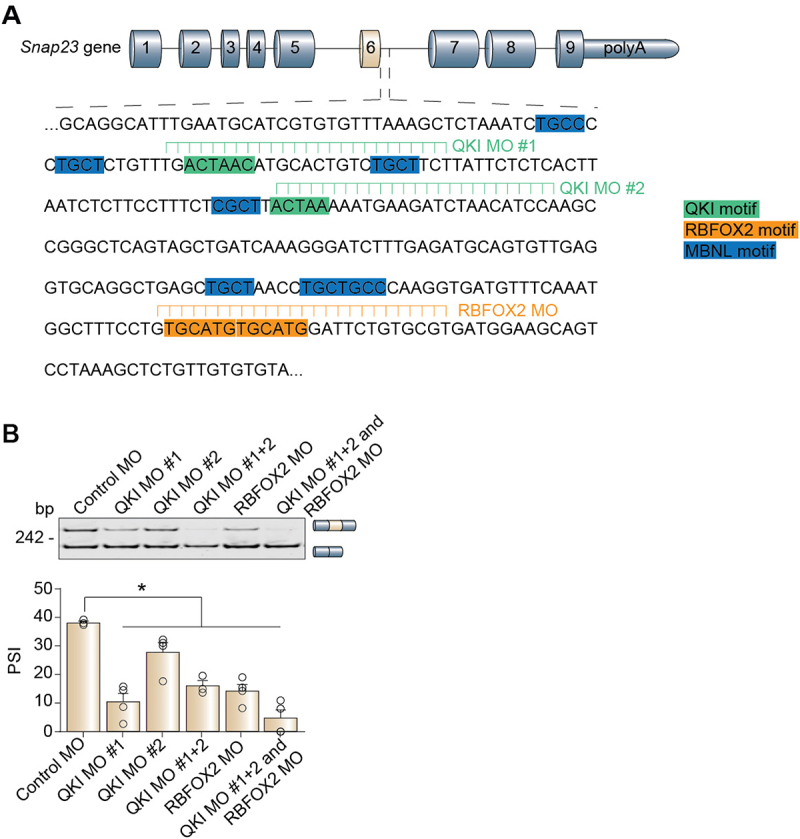
Blocking endogenous QKI and RBFOX2 motifs reduces Snap23 microexon inclusion. (A) Schematic of the RBP motifs blocked using morpholino (MO) antisense oligonucleotides in C2C12 cells. (B) Alternative splicing of the Snap23 microexon in myotubes (differentiated for 4 days) after putative RBP motifs were blocked with the respective MOs was evaluated by RT-PCR assays. The percent spliced-in (PSI) values were calculated by densitometry. Results are shown as the mean ± SEM, *p ≤ 0.05 versus control MO, one-way ANOVA with Dunnett’s multiple comparisons test, *n* = 3–4 independent replicates. bp: base pairs.

A caveat to the minigene study is that there could be distal regulatory elements (>300 base pairs from the splice sites) that are necessary for proper Snap23 microexon splicing which were not cloned into the minigene. To overcome this limitation, we designed morpholino (MO) antisense oligonucleotides to block the endogenous QKI and RBFOX2 binding motifs in C2C12 cells (Figure 8A). When bound to RNA molecules, MOs hide the target regions and prevent the sequences from being recognized by *trans*-acting factors, such as RBPs. When we blocked any combination of the QKI and RBFOX2 motifs, we observed a significant reduction in inclusion of the Snap23 microexon (Figure 8B, lanes 2–5). Notably, blocking all four motifs together had the strongest effect on Snap23 microexon inclusion (Figure 8B, lane 6). Therefore, we concluded that the QKI and RBFOX2 motifs were active, occupied by the RBPs in living cells, and necessary for proper alternative splicing regulation of the Snap23 microexon.

## Discussion

Alternative splicing wields extraordinary power in shaping organ development and tissue identity [5]. Microexons are highly conserved genomic regions that undergo tissue-specific and developmental stage-specific alternative splicing, and microexons often encode in-frame amino acid peptides that confer tissue-specific protein-protein interaction capacities [25]. There are approximately more than 13,000 microexons in the human transcriptome [26], yet only very few have been explored and in a limited number of tissues. Most microexon studies have been concentrated to the brain [23,26,27,80–85]. To our knowledge, almost nothing is known about the regulation of microexons that are tissue-specific for cardiac and skeletal muscles, other than a few reports of microexon splicing in muscle [30–32,86]. This is significant because, together with the brain, striated muscles are the organs with the highest levels of tissue-specific and conserved alternative splicing [4].

Our current study focused on understanding how alternative splicing of a tissue-specific microexon in the *Snap23* gene is regulated. We demonstrated that the Snap23 alternative region aligns with the classical features of a microexon: (1) splicing regulation is tissue-specific and evolutionarily conserved, with inclusion being restricted to adult heart and skeletal muscle tissues in both mice (Figure 1C-D and Figure S1) and humans (Figure S2A), (2) the exon is a multiple of three nucleotides (Figure 1A), and (3) the encoded 11 amino acid peptide is conserved in mammals (Figure S2B).

As a member of the soluble N-ethylmaleimide-sensitive factor attachment protein receptor (SNARE) family, SNAP23 engages two other SNARE proteins during exocytosis to achieve proper vesicle docking and fusion with the plasma membrane [37,87]. According to the SNARE hypothesis, the specificity of cargo transport and membrane fusion is dictated by the interactions between distinct proteins involved in the assembly of the SNARE complex [88,89]. This has been demonstrated for SNAP25, which is the neuronal homolog of SNAP23. The two SNAP25 splice isoforms were shown to form SNARE complexes with the MUNC18–1 and Gβγ accessory proteins with different affinities during exocytosis [90]. Based on previous microexon studies in the brain [26,27], we predict that this microexon would impact the ability of SNAP23 to interact with other proteins. Further studies are needed to elucidate the specific protein binding capabilities conferred by inclusion or skipping of the tissue-specific SNAP23 microexon.

The high prevalence of disrupted alternative splicing programmes in diseased tissues underscores the importance of understanding how splicing regulation occurs during normal development. In striated muscle, mis-splicing is associated with numerous diseases [5,9] including cardiac hypertrophy [91], dilated cardiomyopathy [92], Duchenne muscular dystrophy [93], myotonic dystrophy [94], and spinal muscular atrophy [95]. Heart and skeletal muscle pathologies have also been characterized by errors in the membrane trafficking system [96–98]. We found that inclusion of the Snap23 microexon is strongly downregulated in models of both heart (Figure 2A-E) and skeletal muscle (Figure 2F-H) diseases. Thus, we predict that aberrant SNAP23 isoform expression contributes to the progression of striated muscle diseases.

The prevalence of weak GC splice donors has been shown to increase with genomic complexity, yet less than 1% of annotated splice donor sites recognized by the major spliceosome in humans and mice have the GC sequence [99,100]. This rare class of 5’ splice sites cannot pair with the major spliceosome with perfect complementarity, thus relying upon external factors such as RBPs and enhancer sequences to recruit and stabilize the splicing machinery [101,102]. For these reasons, the GC sites are often enriched at alternatively spliced regions [103,104]. We therefore anticipated the presence of a weak splice site around the Snap23 microexon. Indeed, we found all of the Snap23 splice sites to be highly conserved except for the splice donor site downstream of the microexon (Figure 4A). By introducing a single point mutation to covert the splice donor site from a weak to a strong sequence, we achieved total inclusion of the Snap23 microexon in undifferentiated C2C12 cells (Figure 4C).

We identified QKI and RBFOX2 as the main regulators of Snap23 microexon alternative splicing. During C2C12 cell differentiation, RBFOX2 is the sole RBFOX family member that is expressed, with protein levels significantly upregulated in myotubes compared to myoblasts [33]. Interestingly, QKI protein levels do not change throughout differentiation [33]. Whether the *trans*-acting factors themselves (Figure 5) or their cognate *cis*-regulatory motifs (Figures 7–8) were targeted for removal, we repeatedly observed a decrease in the inclusion levels of the Snap23 microexon. The larger effect observed when QKI and RBFOX2 binding were abrogated together (Figures 5G, 7B, 8B) suggests that these two RBPs cooperate to control alternative splicing of the Snap23 microexon in striated muscle tissues. Notably, RBFOX2 has been previously established to coordinate alternative splicing networks throughout myogenesis [105], and the consensus RBFOX2 binding motif is highly conserved in introns downstream of brain-specific alternative exons in mammals [106]. Furthermore, both QKI and RBFOX2 have been shown to control the inclusion of microexons in the brain [26,107]. The overlapping action of QKI and RBFOX2 in splicing regulation has also been reported in ovarian cancer [108], and global splicing analysis of eight solid cancer varieties has revealed a high overlap of exons that contained both QKI and RBFOX2 motifs in the flanking introns [109].

We were surprised, however, to see that the phenotypes from the QKI motif deletion experiment (Figure 7B) were not consistent with those from the QKI knockdown (Figure 5C) and QKI morpholino (Figure 8B) experiments. It is known that the QKI response element is often bipartite, consisting of the ACUAA(C) core sequence and a half-site motif (UAAY) located approximately 1–20 nucleotides downstream [79]. Yet, some RNA molecules can tolerate variability in the half-site sequence and contain motifs that partially compensate for a missing half-site [79]. Indeed, there is a conserved half-site motif (UAAC) present 12 nucleotides downstream of the QKI #2 core sequence. We suspect that the morpholinos extending downstream of the QKI core sequences are blocking putative half-site motifs to preclude RBP binding. Conversely, the half-site motifs are preserved in the QKI motif deletion experiments and are sufficient for QKI regulation to still occur.

The additional contribution of the MBNL1 and MBNL2 proteins, although minor, highlights the intricate layers of complexity that underly RBP regulation of alternative splicing. Our data showed that the change in Snap23 microexon inclusion observed upon disruption of QKI binding is compounded by depletion of MBNL1 and MBNL2 (Figures 5F, 7C). These findings are in agreement with previous studies demonstrating that MBNL1 and QKI share splicing targets and act together to achieve proper alternative splicing regulation [110,111]. Although not explored in this study, interesting reports have documented a dynamic interplay between MBNL1 and RBFOX2, where these two RBPs can cooperate or compete with each other to govern alternative splicing outcomes [112–114].

Despite the proven importance and disease association of alternative splicing, efforts towards determining the mechanisms underlying alternative splicing regulation have been limited. Collectively, our work adds to a growing number of studies focused on understanding these regulatory systems. We have expanded upon the knowledge in the field by demonstrating that the overlapping action of QKI and RBFOX2 is not limited to cancer but is also an active regulatory mechanism in muscle. We propose a model whereby the MBNL proteins, QKI, and RBFOX2 all contribute to the tight regulation of the Snap23 alternative splicing transition during myogenesis. Uncovering the molecular players that govern splicing programmes is the necessary foundation for potentially targeting these transitions as a therapeutic approach.

## Supplementary Material

SupplementaryMaterial_Figures_Tables_FINAL.docx

## Data Availability

The authors confirm that the data supporting the findings of this study are available within the article and its supplementary materials.
