# Supplementary material for: Alternative splicing of the Snap23 microexon is regulated by MBNL, QKI, and RBFOX2 in a tissue-specific manner and is altered in striated muscle diseases
Source: RNA Biol. 2025 Apr 10;22(1):1–20. doi: 10.1080/15476286.2025.2491160 (PMC12064062; doi:10.1080/15476286.2025.2491160)
Supplement: SupplementaryMaterial_Figures_Tables_FINAL.docx [file KRNB_A_2491160_SM9300.docx]

**Alternative splicing of the Snap23 microexon is regulated by MBNL, QKI, and RBFOX2 in a tissue-specific manner and is mis-spliced in striated muscle diseases**

Gabrielle M. Gentile^1,2^, R. Eric Blue^1^, Grant A. Goda^3^, Bryan B. Guzman^4^, Rachel A. Szymanski^2^, Eunice Y. Lee^1^, Nichlas M. Engels^1^, Emma R. Hinkle^1,2^, Hannah J. Wiedner^1,2^, Aubriana N. Bishop^1^, Jonathan T. Harrison^1^, Hua Zhang^1^, Xander H. T. Wehrens^5^, Daniel Dominguez^4,6,8^, Jimena Giudice^1,2,7,8,*^

^1^Department of Cell Biology and Physiology, The University of North Carolina at Chapel Hill, Chapel Hill, NC 27599, USA. ^2^Curriculum in Genetics and Molecular Biology, The University of North Carolina at Chapel Hill, Chapel Hill, NC 27599, USA. ^3^Department of Chemistry, The University of North Carolina at Chapel Hill, Chapel Hill, NC 27599, USA. ^4^Department of Pharmacology, The University of North Carolina at Chapel Hill, Chapel Hill, NC 27599, USA. ^5^Cardiovascular Research Institute, Baylor College of Medicine, Houston, TX 77030, USA. ^6^Lineberger Comprehensive Cancer Center, The University of North Carolina at Chapel Hill, Chapel Hill, NC 27599, USA. ^7^McAllister Heart Institute, The University of North Carolina at Chapel Hill, Chapel Hill, NC 27599, USA. ^8^RNA Discovery Center, The University of North Carolina at Chapel Hill, Chapel Hill, NC 27599, USA.

*Corresponding author. Mailing address: Department of Cell Biology and Physiology. The University of North Carolina at Chapel Hill. 6340B Medical Biomolecular Research Building. 111 Mason Farm Rd., Chapel Hill, NC 27599, USA. Telephone number: 1-919-962-6260. Email address: [jimena_giudice@med.unc.edu](mailto:jimena_giudice@med.unc.edu)

**
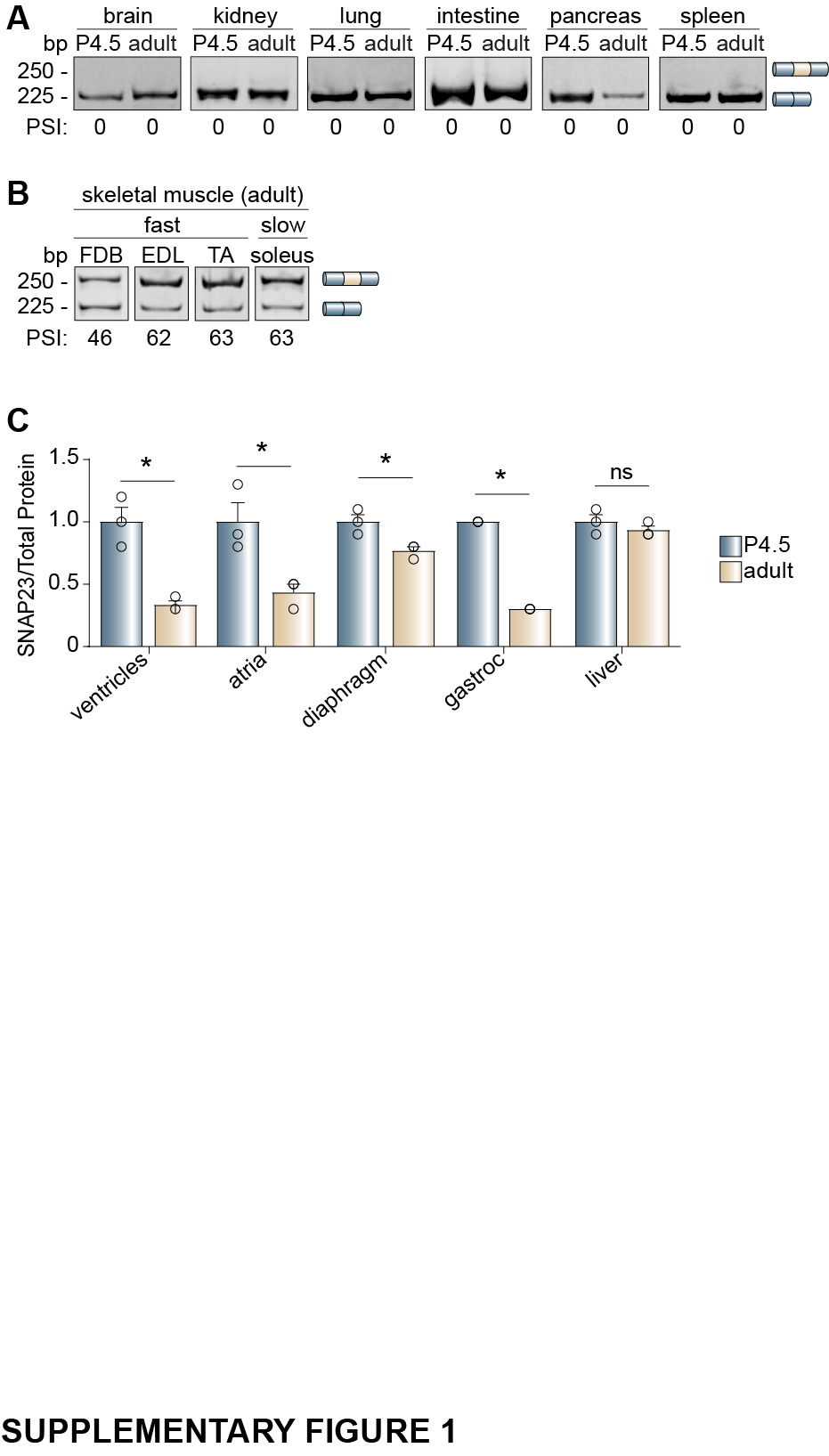
**

**Supplementary Figure 1. Inclusion of the Snap23 microexon is striated muscle-specific. (A)** Alternative splicing of the Snap23 microexon in murine non-striated muscle tissues at postnatal day 4.5 (P4.5) and adulthood (3-4 months old) was evaluated by RT-PCR assays. **(B)** Alternative splicing of the Snap23 microexon in various adult mouse skeletal muscle tissues containing fast-twitch and slow-twitch fibers was evaluated by RT-PCR assays. The percent spliced-in (PSI) values were calculated by densitometry. **(C)** Total SNAP23 protein levels in various mouse tissues at P4.5 and adulthood (3-4 months old) were evaluated by western blot assays. Total SNAP23 protein levels were calculated by densitometry. Results are shown as the mean ± SEM, *p ≤ 0.05 versus P4.5, unpaired t-test with Welch’s correction, *n* = 3 independent replicates. bp: base pairs; EDL: extensor digitorum longus; FDB: flexor digitorum brevis; gastroc: gastrocnemius; ns: not significant; TA: tibialis anterior.

**
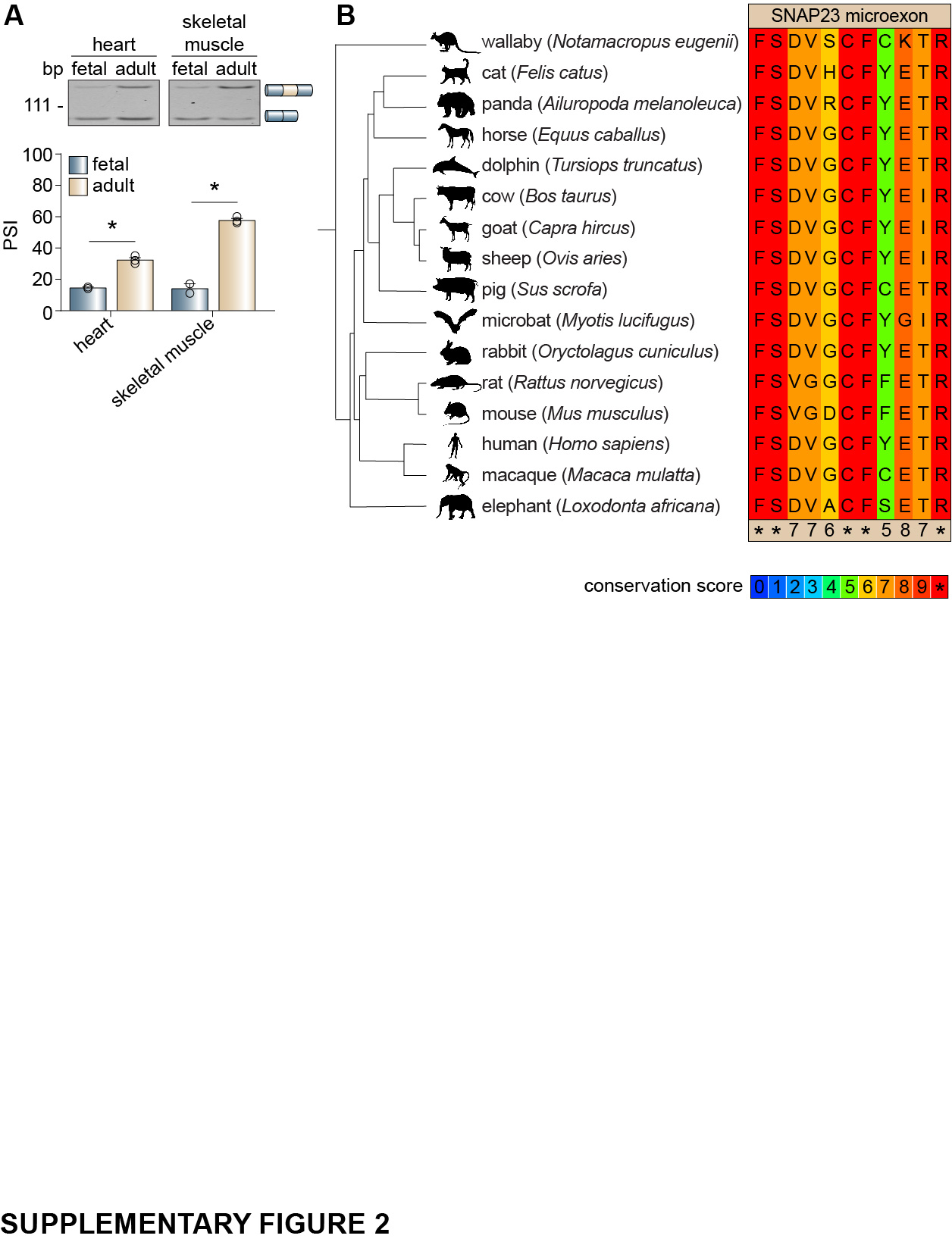
**

**Supplementary Figure 2. Conservation of the Snap23 microexon during mammalian evolution. (A)** Alternative splicing of the SNAP23 microexon in fetal and adult human striated muscle tissues was evaluated by RT-PCR assays. The percent spliced-in (PSI) values were calculated by densitometry. **(B)** Phylogenetic tree of the SNAP23 microexon protein sequence generated using TimeTree 5 following a sequence alignment in PRALINE. Colors indicate the degree of amino acid residue conservation across the represented mammalian species. Results are shown as the mean ± SEM, *p ≤ 0.05 versus fetal tissue, unpaired t-test with Welch’s correction, *n* = 2-3 independent replicates. bp: base pairs.

**
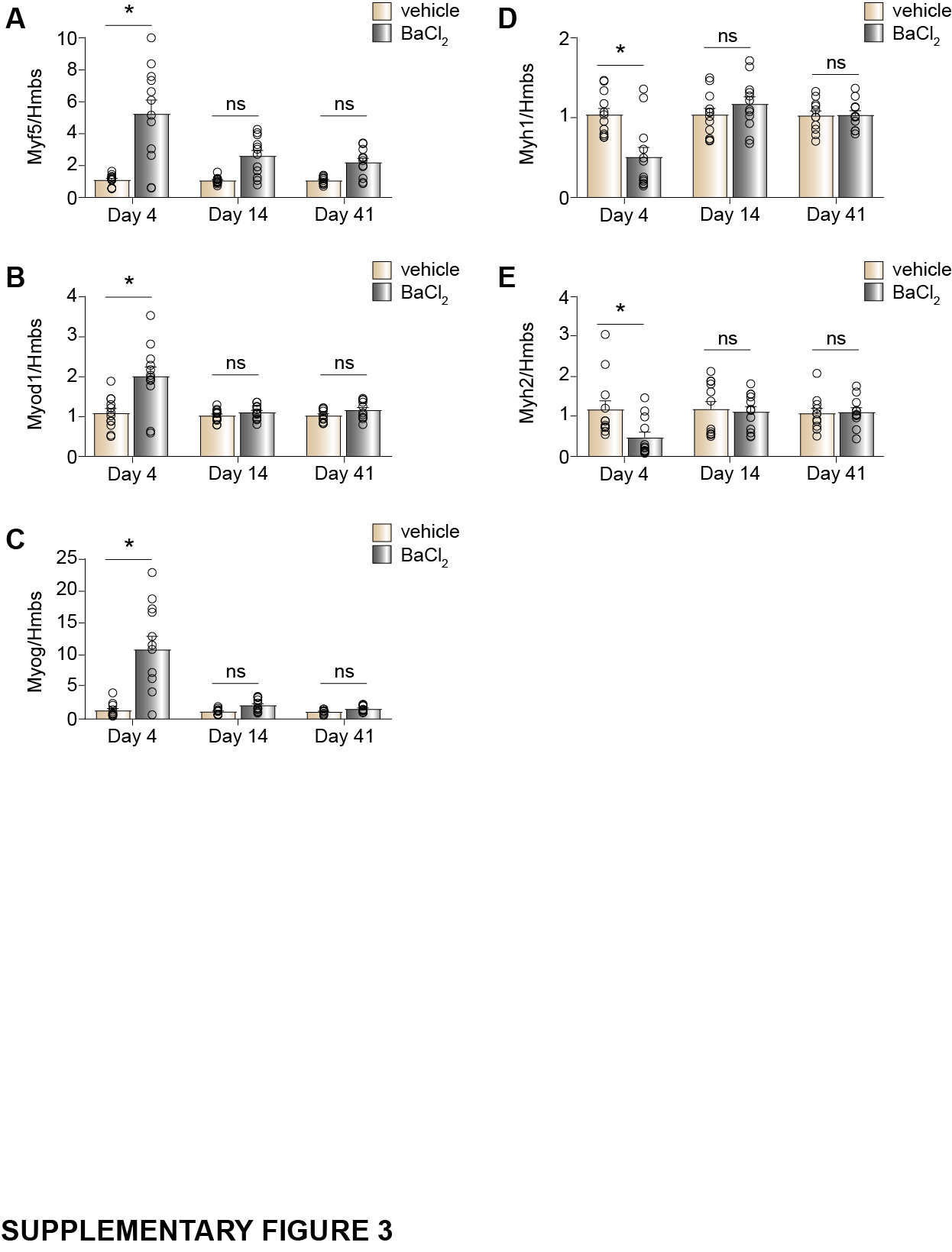
**

**Supplementary Figure 3. Activation of myogenic markers after BaCl_2_ injury in skeletal muscle. (A-E)** The mRNA levels of **(A)** Myf5, **(B)** Myod1, **(C)** Myog, **(D)** Myh1, and **(E)** Myh2 after BaCl_2_ injection of the *tibialis anterior* muscle were evaluated by quantitative real-time PCR (qPCR) assays. Results are shown as the mean ± SEM, *p ≤ 0.05 versus vehicle, two-way ANOVA with Tukey’s multiple comparisons test, *n* = 11-12 independent replicates. Hmbs: hydroxymethylbilane synthase; Myf5: myogenic factor 5; Myh1: myosin, heavy chain 1, skeletal muscle, adult; Myh2: myosin, heavy chain 2, skeletal muscle, adult; Myod1: myogenic differentiation 1; Myog: myogenin. ns: not significant.

**
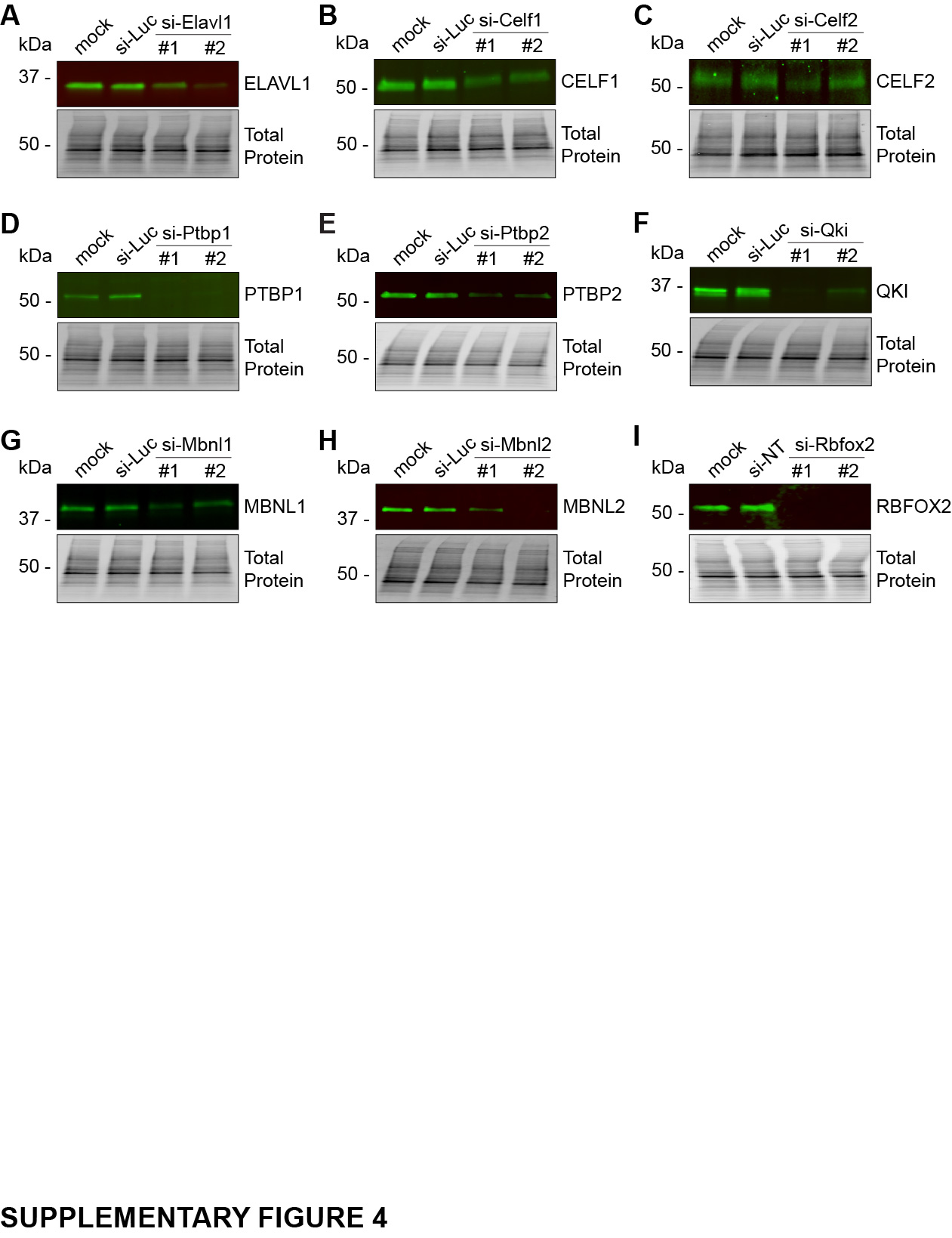
**

**Supplementary Figure 4. Validation of RBP single knockdowns in C2C12 cells. (A-I)** Protein expression following depletion of **(A)** ELAVL1, **(B)** CELF1, **(C)** CELF2, **(D)** PTBP1, **(E)** PTBP2, **(F)** QKI, **(G)** MBNL1, **(H)** MBNL2, and **(I)** RBFOX2 was evaluated by western blot assays. Protein lysates were prepared from either undifferentiated myoblasts (si-Ptbp1, si-Ptbp2) or differentiated myotubes (si-Elavl1, si-Celf1, si-Celf2, si-Qki, si-Mbnl1, si-Mbnl2, si-Rbfox2). CELF: CUGBP Elav-like family member; ELAVL: embryonic lethal, abnormal vision-like; Luc: Luciferase; MBNL: muscleblind-like splicing regulator; NT: Non-Targeting; PTBP: polypyrimidine tract binding protein; QKI: quaking; RBFOX: RNA binding fox-1 homolog.

**
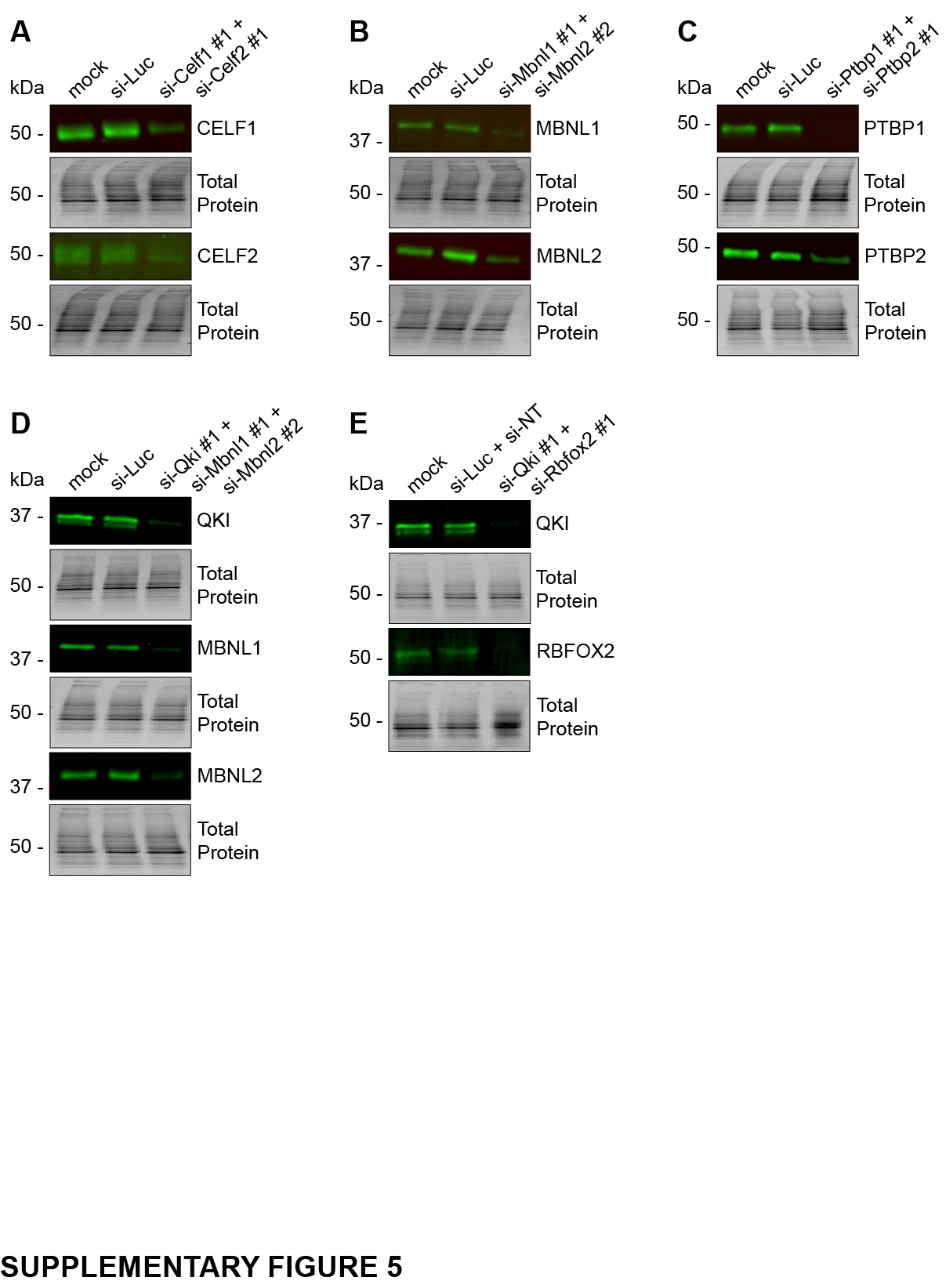
**

**Supplementary Figure 5. Validation of RBP combination knockdowns in C2C12 cells. (A-E)** Protein expression following depletion of **(A)** CELF1 + CELF2, **(B)** MBNL1 + MBNL2, **(C)** PTBP1 + PTBP2, **(D)** QKI + MBNL1 + MBNL2, and **(E)** QKI + RBFOX2 was evaluated by western blot assays. Protein lysates were prepared from either undifferentiated myoblasts (si-Ptbp1 + si-Ptbp2) or differentiated myotubes (si-Celf1 + si-Celf2, si-Mbnl1 + si-Mbnl2, si-Qki + si-Mbnl1 + si-Mbnl2, si-Qki + si-Rbfox2). CELF: CUGBP Elav-like family member; Luc: Luciferase; MBNL: muscleblind-like splicing regulator; NT: Non-Targeting; PTBP: polypyrimidine tract binding protein; QKI: quaking; RBFOX: RNA binding fox-1 homolog.

**
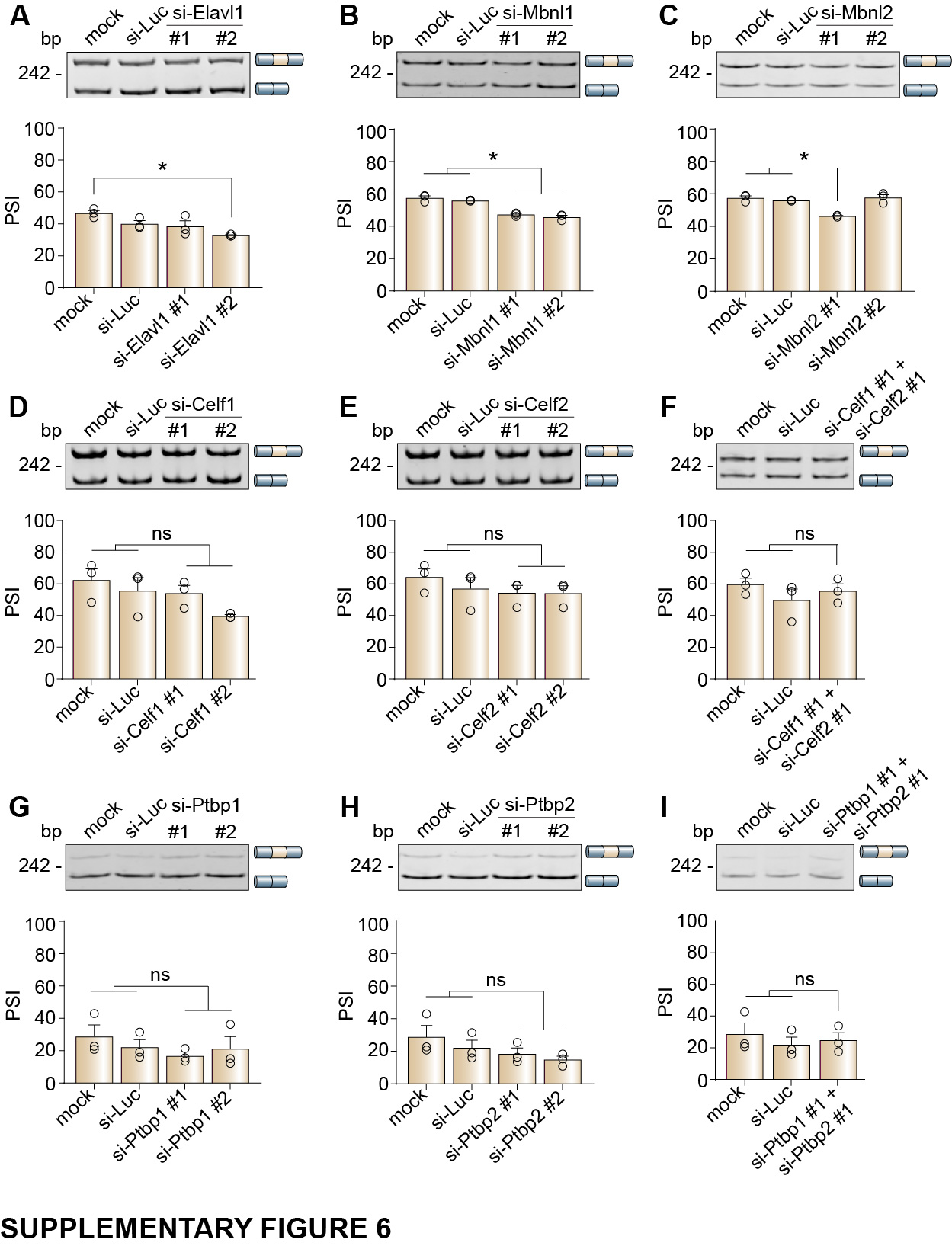
**

**Supplementary Figure 6. Alternative splicing of the Snap23 microexon in response to RBP depletions. (A-I)** Alternative splicing of the Snap23 microexon following depletion of **(A)** ELAVL1, **(B)** MBNL1, **(C)** MBNL2, **(D)** CELF1, **(E)** CELF2, **(F)** CELF1 + CELF2, **(G)** PTBP1, **(H)** PTBP2, and **(I)** PTBP1 + PTBP2 was evaluated by RT-PCR assays. Two independent si-RNAs were used for each RBP. The percent spliced-in (PSI) values were calculated by densitometry. Results are shown as the mean ± SEM, *p ≤ 0.05 versus mock and si-Luc conditions, ordinary one-way ANOVA with Tukey’s multiple comparisons test, *n* = 3 independent replicates. bp: base pairs; CELF: CUGBP Elav-like family member; ELAVL: embryonic lethal, abnormal vision-like; Luc: Luciferase; MBNL: muscleblind-like splicing regulator; ns: not significant; PTBP: polypyrimidine tract binding protein.

**
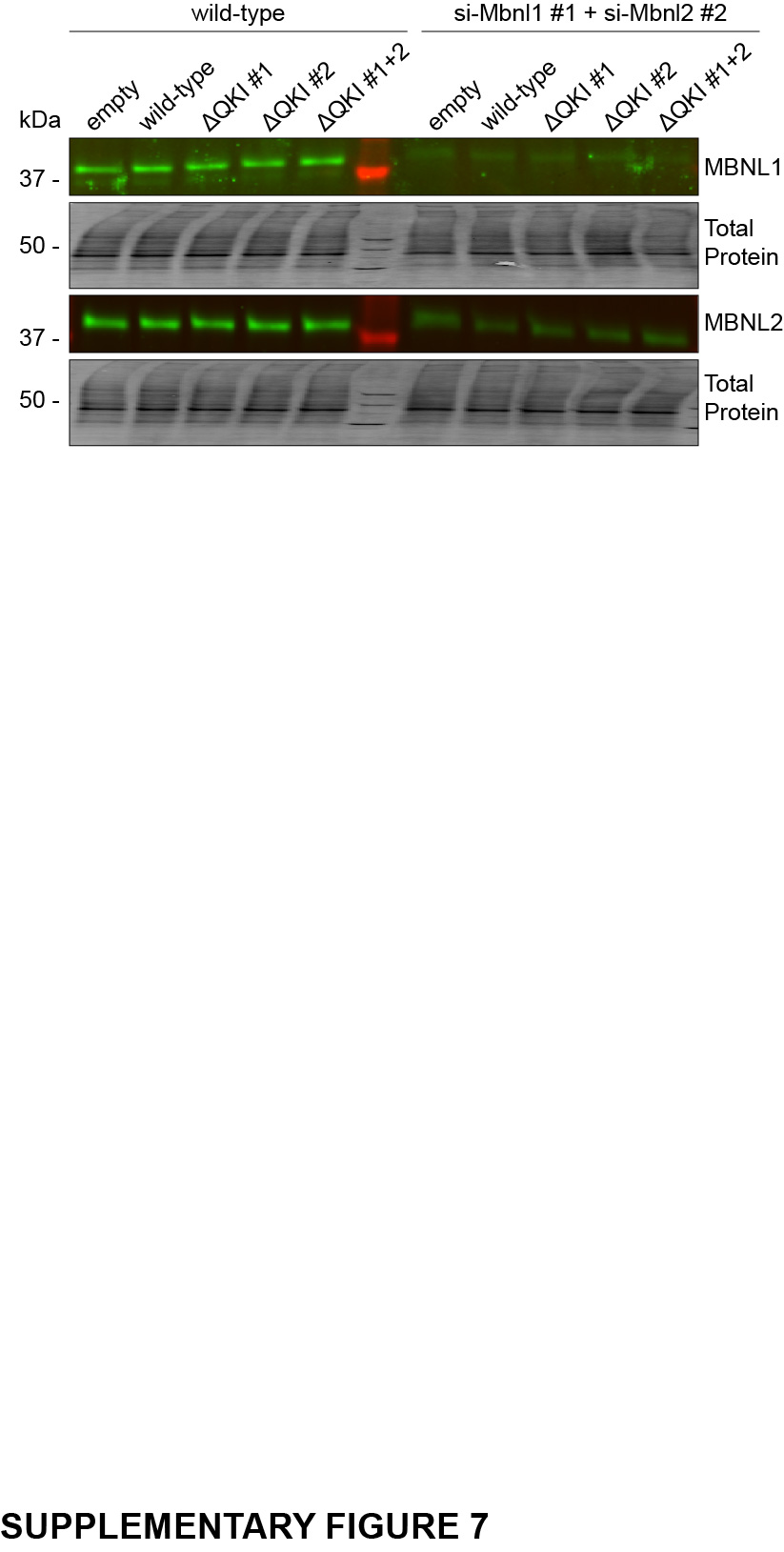
**

**Supplementary Figure 7. Validation of MBNL1 + MBNL2 knockdowns in C2C12 cells expressing the Snap23 minigenes.** Protein expression following depletion of MBNL1 + MBNL2 was evaluated by western blot assays. Protein lysates were prepared from differentiated myotubes. MBNL: muscleblind-like splicing regulator.

**SUPPLEMENTARY TABLE 1**

| **Name** | **Company** | **si-RNA ID** | **Sequence (5’ to 3’)** |
| --- | --- | --- | --- |
| si-Luciferase | Invitrogen | #465377 | GCACUCUGAUUGACAAAUACGAUUU |
| si-Celf1 #1 |  | MSS203372 | GGACAGAUUGAAGAGUGCCGGAUAU |
| si-Celf1 #2 |  | MSS203374 | CCAUGAACGGCUUUCAAAUUGGAAU |
| si-Celf2 #1 |  | MSS274200 | CAGAGUAAAGGUUGUUGUUUCGUAA |
| si-Celf2 #2 |  | MSS204012 | GCUGGAGCCACUGUCGGAUUGAAUA |
| si-Elavl1 #1 |  | MSS205313 | GGGUUGCCUUUAUCCGGUUUGACAA |
| si-Elavl1 #2 |  | MSS205314 | UGGCCAUAGCAAGUCUGAACGGCUA |
| si-Mbnl1 #1 |  | MSS226392 | CCACAGCCAACCAGAUACCCAUAAU |
| si-Mbnl1 #2 |  | MSS226393 | GCAUUUCUCCCACCAGGCUCAAUAU |
| si-Mbnl2 #1 |  | MSS200587 | GCGUUGCAUGAGGGAGAAAUGCAAA |
| si-Mbnl2 #2 |  | MSS272396 | GAGAUUAAUGGGAGGAACAAUUUGA |
| si-Ptbp1 #1 |  | MSS276537 | GGUGUGGUCAAAGGCUUCAAGUUCU |
| si-Ptbp1 #2 |  | MSS276539 | CCUCUGGAGACAGCCAGCCUUCACU |
| si-Ptbp2 #1 |  | MSS225938 | GGUGGCAAUACAGUCCUGUUGGUUA |
| si-Ptbp2 #2 |  | MSS225940 | GGGCACUGUGAAAGCAUUUAAGUUU |
| si-Qki #1 |  | MSS208338 | GAGCGGUUGAAGAAGUGAAGAAGUU |
| si-Qki #2 |  | MSS276677 | GAGCGGCUGCUGGACGAAGAAAUUA |
| si-Non-Targeting | Dharmacon | D-001210-03 | AUGUAUUGGCCUGUAUUAG |
| si-Rbfox2 #1 |  | D-051552-01 | GCAAAUGGCUGGAAGUUAA |
| si-Rbfox2 #2 |  | D-051552-04 | CGAGAAUAGUGCUGAUGCA |

**Supplementary Table 1.** Sequence of the small interfering RNAs (si-RNAs) used for RNA-binding protein (RBP) knockdown experiments. Celf: CUGBP Elav-like family member; Elavl: embryonic lethal, abnormal vision-like; Mbnl: muscleblind-like splicing regulator; Ptbp: polypyrimidine tract binding protein; Qki: quaking; Rbfox: RNA binding fox-1 homolog.

**SUPPLEMENTARY TABLE 2**

| **Gene symbol** | **Probe ID** | **Species** | **Exon boundary** | **Amplicon length (nt)** |
| --- | --- | --- | --- | --- |
| *Hmbs* | Mm01143545_m1 | mouse | 6 – 7 | 81 |
| *Myf5* | Mm00435125_m1 |  | 2 – 3 | 71 |
| *Myh1* | Mm01332489_m1 |  | 27 – 28 | 104 |
| *Myh2* | Mm01332564_m1 |  | 39 – 40 | 103 |
| *Myod1* | Mm01203489_g1 |  | 2 – 3 | 103 |
| *Myog* | Mm00446194_m1 |  | 1 – 2 | 69 |

**Supplementary Table 2.** TaqMan® probes (Thermo Fisher Scientific) used for qPCR analysis. Hmbs: hydroxymethylbilane synthase; Myf5: myogenic factor 5; Myh1: myosin, heavy chain 1, skeletal muscle, adult; Myh2: myosin, heavy chain 2, skeletal muscle, adult; Myod1: myogenic differentiation 1; Myog: myogenin; nt: nucleotides.

**SUPPLEMENTARY TABLE 3**

| **Antibody** | **Catalog** | **Company** | **Host** | **Clone** | **Dilution** |
| --- | --- | --- | --- | --- | --- |
| CELF1 | sc-20003 | Santa Cruz Biotechnology | mouse | monoclonal | 1:200 |
| CELF2 | sc-47731 | Santa Cruz Biotechnology | mouse | monoclonal | 1:200 |
| ELAVL1 | 11910-1-AP | Proteintech | rabbit | polyclonal | 1:2,000 |
| MBNL1 | sc-515374 | Santa Cruz Biotechnology | mouse | monoclonal | 1:500 |
| MBNL2 | sc-136167 | Santa Cruz Biotechnology | mouse | monoclonal | 1:500 |
| MYH3 | sc-53091 | Santa Cruz Biotechnology | mouse | monoclonal | 1:200 |
| PTBP1 | ab133734 | Abcam | rabbit | monoclonal | 1:10,000 |
| PTBP2 | ab154787 | Abcam | rabbit | monoclonal | 1:500 |
| QKI | ab126742 | Abcam | rabbit | monoclonal | 1:1,000 |
| RBFOX2 | sc-271407 | Santa Cruz Biotechnology | mouse | monoclonal | 1:500 |
| SNAP23 | ab3340 | Abcam | rabbit | polyclonal | 1:1,000 |

**Supplementary Table 3.** Antibodies used for western blot assays. CELF: CUGBP Elav-like family member; ELAVL: embryonic lethal, abnormal vision-like; MBNL: muscleblind-like splicing regulator; MYH: myosin heavy chain; PTBP: polypyrimidine tract binding protein; QKI: quaking; RBFOX: RNA binding fox-1 homolog; SNAP: synaptosome-associated protein.

**SUPPLEMENTARY TABLE 4**

| **Probe** | **Sequence** | **6-FAM location** | **Company** |
| --- | --- | --- | --- |
| QKI #1 | UUUGACUAACAUGCACUGU | 3’ end | IDT |
| QKI #2 | GCUUACUAAAAAUGAAGAU | 3’ end |  |
| RBFOX2 #1 | AAUGGCUUUCCUGUGCAUG | 5’ end |  |
| RBFOX2 #2 | UGCAUGGAUUCUGUGCGUGA | 3’ end |  |

**Supplementary Table 4.** Probes used for fluorescence polarization assays. QKI: quaking; RBFOX: RNA binding fox-1 homolog.

**SUPPLEMENTARY TABLE 5**

| **Plasmid** | **Primer** | **Sequence (5’ to 3’)** | **Ta (°C)** | **PCR substrate** |
| --- | --- | --- | --- | --- |
| wild-type | Fwd | ATATATGTCGACaatactcgggaagacgag | 58 | C2C12 cell DNA |
|  | Rev | ATATATACTAGTtacacacaacagagctttaggactg |  |  |
| ΔQKI #1 | Fwd | ATGCACTGTCTGCTTCTTATTC | 64 | wild-type plasmid |
|  | Rev | CAAACAGAGCAGGGGCAG |  |  |
| ΔQKI #2 | Fwd | AAATGAAGATCTAACATCCAAG | 58 | wild-type plasmid |
|  | Rev | AAGCGAGAAAGGAAGAGATTAAG |  |  |
| ΔQKI #1+2 | Fwd | same as used for ΔQKI #2 plasmid |  | ΔQKI #1 plasmid |
|  | Rev |  |  |  |
| ΔRBFOX2 #1 | Fwd | TGCATGGATTCTGTGCGT | 63 | wild-type plasmid |
|  | Rev | CAGGAAAGCCATTTGAAACATC |  |  |
| ΔRBFOX2 #1+2 | Fwd | GATTCTGTGCGTGATGGAAG | 64 | wild-type plasmid |
|  | Rev | same as used for ΔRBFOX2 #1 plasmid |  |  |
| ΔQKI #1+2; ΔRBFOX2 #1+2 | Fwd | same as used for ΔRBFOX2 #1+2 plasmid |  | ΔQKI #1+2 plasmid |
|  | Rev |  |  |  |
| splice donor C>T transition | Fwd | TTGAAACCAGGTAGGCATTTGAATG | 63 | wild-type plasmid |
|  | Rev | AGAAACAGTCACCAACTGAG |  |  |
| all plasmids | Fwd | AGGGCGAGCAAAATCCCTTT | n/a | for Sanger sequencing |
|  | Rev | CCCCTGCTGGCAAATGAGAT |  |  |

**Supplementary Table 5.** Forward (Fwd) and reverse (Rev) primers used for minigene cloning and sequencing. Ta: annealing temperature.

**SUPPLEMENTARY TABLE 6**

| **Plasmid** | **Insert Description** | **Shorthand Name** | **Backbone** | **Promoter** | **Antibiotic Resistance** |
| --- | --- | --- | --- | --- | --- |
| wild-type | Snap23-ex33nts | pGG45 | RHCglo | RSV | Ampicillin |
| ΔQKI #1 | Snap23-ex33nts  ΔQKI core #1 | pGG49 |  |  |  |
| ΔQKI #2 | Snap23-ex33nts  ΔQKI core #2 | pGG65 |  |  |  |
| ΔQKI #1+2 | Snap23-ex33nts  ΔQKI core #1+2 | pGG66 |  |  |  |
| ΔRBFOX2 #1 | Snap23-ex33nts  ΔRBFOX2 #1 | pGG67 |  |  |  |
| ΔRBFOX2 #1+2 | Snap23-ex33nts  ΔRBFOX2 #1+2 | pGG69 |  |  |  |
| ΔQKI #1+2; ΔRBFOX2 #1+2 | Snap23-ex33nts  ΔQKI #1+2 and  ΔRBFOX2 #1+2 | pGG74 |  |  |  |
| splice donor C>T transition | Snap23-ex33nts  sub-SpliceDonor-CtoT | pGG90 |  |  |  |

**Supplementary Table 6.** Description of minigene plasmids. RSV: Rous Sarcoma Virus.

**SUPPLEMENTARY TABLE 7**

| **Morpholino** | **Sequence** | **Company** |
| --- | --- | --- |
| QKI #1 | AGAAGCAGACAGTGCATGTTAGTCA | Gene Tools |
| QKI #2 | TGGATGTTAGATCTTCATTTTTAGT |  |
| RBFOX2 #1+2 | ACGCACAGAATCCATGCACATGCAC |  |

**Supplementary Table 7.** Morpholino antisense oligonucleotides used for blocking putative RNA-binding protein motifs. QKI: quaking; RBFOX: RNA binding fox-1 homolog.
